# FHR4‐based immunoconjugates direct complement‐dependent cytotoxicity and phagocytosis towards HER2‐positive cancer cells

**DOI:** 10.1002/1878-0261.12554

**Published:** 2019-09-30

**Authors:** Carole Seguin‐Devaux, Jean‐Marc Plesseria, Charlène Verschueren, Cécile Masquelier, Gilles Iserentant, Marie Fullana, Mihály Józsi, Jacques H. M. Cohen, Xavier Dervillez

**Affiliations:** ^1^ Department of Infection and Immunity Luxembourg Institute of Health Esch‐sur‐Alzette Luxembourg; ^2^ Société d’Accélération des Transferts de Technologies du Nord Direction Territoriale Reims Reims France; ^3^ Complement Research Group, Department of Immunology ELTE Eötvös Loránd University Budapest Hungary; ^4^ LRN EA4682 Université Reims Champagne Ardenne (URCA) France; ^5^ Life Sciences Research Unit (LSRU), Signal Transduction Laboratory University of Luxembourg Belvaux Luxembourg

**Keywords:** C4bp, CDC, complement resistance, FHR4, MAC, multimers

## Abstract

Directing selective complement activation towards tumour cells is an attractive strategy to promote their elimination. In the present work, we have generated heteromultimeric immunoconjugates that selectively activate the complement alternative pathway (AP) on tumour cells. We used the C4b‐binding protein C‐terminal‐α‐/β‐chain scaffold for multimerisation to generate heteromultimeric immunoconjugates displaying (a) a multivalent‐positive regulator of the AP, the human factor H‐related protein 4 (FHR4) with; (b) a multivalent targeting function directed against erbB2 (HER2); and (c) a monovalent enhanced GFP tracking function. Two distinct V_H_H targeting two different epitopes against HER2 and competing either with trastuzumab or with pertuzumab‐recognising epitopes [V_H_H(T) or V_H_H(P)], respectively, were used as HER2 anchoring moieties. Optimised high‐FHR4 valence heteromultimeric immunoconjugates [FHR4/V_H_H(T) or FHR4/V_H_H(P)] were selected by sequential cell cloning and a selective multistep His‐Trap purification. Optimised FHR4‐heteromultimeric immunoconjugates successfully overcame FH‐mediated complement inhibition threshold, causing increased C3b deposition on SK‐OV‐3, BT474 and SK‐BR3 tumour cells, and increased formation of lytic membrane attack complex densities and complement‐dependent cytotoxicity (CDC). CDC varies according to the pattern expression and densities of membrane‐anchored complement regulatory proteins on tumour cell surfaces. In addition, opsonised BT474 tumour cells were efficiently phagocytosed by macrophages through complement‐dependent cell‐mediated cytotoxicity. We showed that the degree of FHR4‐multivalency within the multimeric immunoconjugates was the key element to efficiently compete and deregulate FH and FH‐mediated convertase decay locally on tumour cell surface. FHR4 can thus represent a novel therapeutic molecule, when expressed as a multimeric entity and associated with an anchoring system, to locally shift the complement steady‐state towards activation on tumour cell surface.

## Graphical abstract

We propose a new approach of complement‐mediated destructive tumour cell targeting by generating immunoconjugates harbouring multimeric (a) factor H‐related protein 4 (FHR4)‐complement effector functions with; (b) V_H_H anti‐HER2 targeting functions. The number of FHR4 valences dictates the efficacy of the multimers to selectively and locally activate complement alternative pathway on HER2‐tumour cell surface, leading to complement‐dependent cytotoxicity and complement‐dependent cell‐mediated phagocytosis.

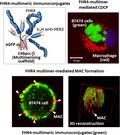

**Abbreviations**
∆C1qHS: C1q‐deficient human serum
∆C5HS: C5‐depleted human serum
∆FBHS: factor B‐deficient human serum
ADCC: antibody‐dependent cell‐mediated cytotoxicity
ADCP: antibody-dependent cell-mediated phagocytosis
AP: complement alternative pathway
C4bp: C4b‐binding protein
CD46: membrane cofactor protein
CD55: decay accelerating factor
CD59: protectin
CDC: complement‐dependent cytotoxicity
CDCC: complement‐dependent cell‐mediated cytotoxicity
CDCP: complement‐dependent cell‐mediated phagocytosis
CIT: complement inhibitory threshold
CP: complement classical pathway
EFF: AP‐effector function
eGFP: enhanced GFP
FB: factor B
FD: factor D
FH: factor H
FHR4: factor H‐related protein 4
FI: factor I
GVB^+^: gelatin veronal buffer containing MgCl_2_ and EGTA (without CaCl_2_)
GVB^++^: gelatin veronal buffer containing CaCl_2_ and MgCl_2_
MAC: membrane attack complex
mCRPs: membrane‐associated complement regulatory proteins
MFI: mean fluorescence intensity
NHS: normal human serum
PFA: paraformaldehyde
RBC: red blood cells
SCR: short consensus repeat
SRS: SYPRO ruby staining
TaF: targeting function
TrF: tracking function
V_H_H(T) or V_H_H(P): trastuzumab‐ or pertuzumab‐competing V_H_H anti‐HER2

## 1 Introduction

The complement system is a major humoral arm of innate immunity (Morgan and Kavanagh, 2018; Reis *et al.*, 2019), acting as a fast and efficient immune surveillance system that can discriminate among healthy host tissues, cellular debris, apoptotic cells and foreign intruders. Complement can be rapidly activated against invading pathogens, either directly through activation of the complement alternative pathway (AP) or indirectly through the activation of the complement classical pathway (CP) or complement lectin pathway (LP). Sequential events of the complement‐directed inflammatory cascades lead to the generation of C3 and C5 convertases that orchestrate the assembly of membrane attack complex (MAC) C5b‐9, leading to direct complement‐dependent cytotoxicity (CDC) and subsequent cell killing (Bubeck, 2014). Moreover, C3b degradation products on opsonised targets promote the activation of phagocytic effector cells and of cytotoxic cells, such as natural killer (NK) cells, *via* CR1 (CD35), CR3 (CD11b/CD18) and CR4 (CD11c/CD18) receptors, leading to complement‐dependent cell‐mediated phagocytosis (CDCP) and complement‐dependent cell‐mediated cytotoxicity (CDCC) (Gelderman *et al.*, 2004). Directing the selective complement activation towards unwanted cells to promote destructive cell targeting is an attractive strategy (Carter and Lieber, 2014), activating AP within few minutes and further promoting CDC and/or CDCC and (Gelderman *et al.*, 2004).

The first approach to modulate complement was established with the development of monoclonal antibodies (mAbs), promoting antibody‐dependent cell‐mediated cytotoxicity (ADCC) and CDC for many types of tumours (Meyer *et al.*, 2014). Although the therapeutic potential for a large majority of antibodies is mainly based on ADCC, contribution of CDC remains controversial (Derer *et al.*, 2014). Four known therapeutic mAbs (i.e., alemtuzumab, cetuximab, ofatumumab and rituximab) induce efficient CDC and opsonisation on B‐cell lymphomas in chronic lymphocytic leukaemia (CLL) (Diebolder *et al.*, 2014; Gancz and Fishelson, 2009; Gorter and Meri, 1999; Idusogie *et al.*, 2001; Richards *et al.*, 2008; Zent, 2008; Zent *et al.*, 2016). The efficiency of mAb‐mediated CDC to kill tumour cells *in vivo* is less clear with solid tumours. Overexpression of membrane complement regulatory proteins (mCRPs) such as membrane cofactor protein (CD46), decay accelerating factor (CD55) and protectin (CD59) is considered to be one of the critical mechanisms by which solid tumours can resist CDC (Gancz and Fishelson, 2009; Golay *et al.*, 2001; Golay *et al.*, 2000). Substantial amounts of research have focused on engineering Fc variant antibodies to improve the capacity of antibodies to promote CDC and/or ADCC or ADCP (Diebolder *et al.*, 2014; Idusogie *et al.*, 2001; Richards *et al.*, 2008). The clinical efficacy of such engineered antibodies remains to be determined in clinical trials.

Several approaches aiming at focusing C3b deposition on targets to mediate CDC have been proposed (Elvington *et al.*, 2012; Kontermann *et al.*, 1997; Shaughnessy *et al.*, 2014). Factor H (FH) is the main regulator of the initial activation of the AP, which is essential in restricting the action of complement to activating surfaces (Ferreira *et al.*, 2010; Kopp *et al.*, 2012). FH maintains the density of C3b molecules on host surfaces below a critical threshold to control C3b amplification and prevent the occurrence of complement‐mediated tissue damage (Jozsi *et al.*, 2015). FH‐related protein 4 (FHR4) was recently described as a positive regulator of the complement AP (Hebecker and Jozsi, 2012). FHR4, like FHR3, recruits C3b to a cell surface by competing with FH, thus promoting immune activation by acting as an antagonist of FH (Caesar *et al.*, 2014; Hebecker and Jozsi, 2012). FHR4 shares sequence homologies with FH short consensus repeat (SCR) domains 6, 8, 9, 19 and 20, which display two C3b binding sites, but is unable to inactivate C3b because of the lack of the FH C3b‐regulatory domains (Jozsi *et al.*, 2015). Importantly, FHR4‐bound C3b favours the subsequent binding of factor B (FB) and properdin to generate FHR4‐C3bBb convertases that are resistant to FH–mediated decay (Hebecker and Jozsi, 2012). Therefore, FHR4 acts as an enhancer of opsonisation and facilitates the recognition of dying cells (Hebecker and Jozsi, 2012; Mihlan *et al.*, 2009) or pathogens by the host’s immune surveillance system for further elimination by direct cell killing or by phagocytosis.

We hypothesised that a multimeric recombinant FHR4 molecule, combined to a targeting moiety recognising a tumour‐associated antigen, could selectively accumulate on target tumour cells, enhancing the local stoichiometry of FHR4, thereby allowing the tumour cell‐established complement inhibition threshold (CIT) to be overcome. We used the C4b‐binding protein (C4bp) C‐terminal of the α and β chains as a scaffold for multimerisation (Dervillez *et al.*, 2006; Oudin *et al.*, 2000) to generate trifunctional heteromultimers harbouring (a) multivalent FHR4; (b) multivalent V_H_H against HER2; and (c) a monovalent enhanced GFP (eGFP) tracking function (TrF). In the present work, we investigated the mode of action of the immunoconjugates *in vitro* against HER2‐tumour cells. We showed that optimised immunoconjugates expressing high FHR4 valences were the most potent immunoconjugates to activate AP and subsequently induce massive C3b deposition, MAC binding and CDC of SK‐OV‐3, BT474 and SK‐BR3 HER2‐overexpressing tumour cell lines, as well as complement‐mediated phagocytosis.

## 2 Materials and methods

### 2.1 Cells and antibodies

All multimers were generated from stable transfected HEK293 cells (ATCC CRL‐1573, Manassas, VA, USA) cultured with Dulbecco’s modified Eagle’s medium (Westburg, Leusden, the Netherlands) supplemented with 10% heat‐inactivated FBS (Life Technologies Europe BV, Merelbeke, Belgium), 1 U·mL^−1^ of penicillin, 1 μg·mL^−1^ of streptomycin (Wesburg) and 4 mm of glutamine (Westburg). BT474 (HTB‐20), SK‐OV‐3 (HTB‐77) and SK‐BR‐3 (HTB‐30) cells were kindly provided by M. Kirschfink (University of Heidelberg). Rabbit anti‐6‐His and goat anti‐ enterokinase cleavage site (DDDDK) polyclonal antibodies (pAbs) were purchased from Bethyl (ImTec Diagnostic NV, Antwerpen, Belgium). A mouse anti‐human FHR4 mAb was purchased from R&D Systems Europe Ltd (Bio‐Techne, Abingdon, UK). Mouse anti‐human C3b/iC3b (Clone 7C12) mAb, unconjugated or phycoerythrin (PE) conjugated, was purchased from CEDARLANE (Sanbio B.V., Uden, the Netherlands). The mouse anti‐human C4d monoclonal antibody, the FB‐depleted, C1q‐depleted and C5‐depleted human sera [FB‐deficient human serum (∆FBHS), C1q‐deficient human serum (∆C1qHS) and C5‐depleted human serum (∆C5HS), respectively] were purchased from Quidel (TECOmedical Benelux BV, Utrecht, the Netherlands). The following antibodies were purchased from ABCAM (Cambridge, UK): mouse anti‐human C5b‐9 (Clone aE11) mAb and AF647‐conjugated donkey anti‐goat immunoglobulin G (IgG) pAb. AF647‐conjugated goat anti‐rabbit IgG pAb was purchased from Invitrogen (Thermo Fisher Scientific BVBA, Merelbeke, Belgium). PE‐conjugated donkey anti‐rabbit IgG pAb was provided by eBioscience (Affymetrix, Rennes, France). Allophycocyanin (APC)‐conjugated goat anti‐mouse IgG was purchased from Jackson ImmunoResearch (Sanbio). PKH26 red fluorescent cell linker was provided by Sigma‐Aldrich (Overijse, Belgium). Propidium iodide (PI), carboxyfluorescein succinimidyl ester (CFSE) cell tracer, 4′,6‐diamidino‐2‐phenylindole dihydrochloride (DAPI) were from Life Technologies (Europe BV). Purified C3b and FH were purchased from Merck KGaA (Darmstadt, Germany). Trastuzumab (Herceptin) and pertuzumab (Perjeta) therapeutic antibodies were obtained from Roche (Prophac, Howald, Luxembourg). The PE‐ or APC‐conjugated mouse anti‐human IgG was from BD Pharmingen (Becton Dickinson Benelux NV, Erembodegem, Belgium). The rabbit anti‐mouse IgG horseradish peroxidase (HRP) and goat anti‐rabbit IgG HRP‐conjugated antibodies were from Sigma‐Aldrich. Mouse anti‐human CD46 AF647‐conjugated IgG1 (Clone MEM‐258) and mouse anti‐human CD55 R‐phycoerythrin (RPE)‐conjugated IgG1 (Clone 67) were from Bio‐Rad (Bio‐Rad Laboratories NV, Temse, Belgium). The mouse anti‐human CD59 fluorescein isothiocyanate (FITC)‐conjugated IgG2a (Clone MEM‐43) was from ImmunoTools GmbH (Friesoythe, Germany). The goat anti‐mouse IgG AF546‐conjugated secondary antibody was from Thermo Fisher Scientific BVBA. Human recombinant macrophage colony‐stimulating factor (M‐CSF) was from Enzo Life Sciences BVBA (Antwerpen, Belgium). Sheep erythrocytes (50% blood suspension in Alsever buffer) and rabbit haemolytic anti‐sheep erythrocyte serum (haemolysin) were purchased from Virion\Serion (Serion Immunologics, Würtzburg, Germany).

### 2.2 Establishment of expression cassettes for multifunctional heteromultimers

The backbone bi‐cistronic pEF‐IRESpac expression vector was used to clone all expression cassettes between EcoRI and XbaI restriction sites of its multiple cloning sites (Hobbs *et al.*, 1998). The signal peptide cloned between EcoRI and Bgl2 is from the tumour necrosis factor receptor superfamily member 16 (UniProt no. http://www.uniprot.org/uniprot/P08138) (Hildinger *et al.*, 2001). cDNA encoding human FHR4A (UniProt no. http://www.uniprot.org/uniprot/Q92496), modified 2D3 and 47D5 V_H_H anti‐HER2 (2D3 V_H_H recognising trastuzumab‐competing epitope or 47D5 V_H_H recognising pertuzumab‐competing epitope, adapted from patent WO2008068280) and eGFP were synthesised (ProteoGenix SAS, Schiltigheim, France) between the Bgl2 and BspE1 restrictions sites. cDNA encoding human C4bp C‐terminal α‐chain (UniProt no. http://www.uniprot.org/uniprot/P04003, aa: 540–597) and the third complement control protein (CCP3 or SCR3) plus the C‐terminal end of the human C4bp β‐chains (UniProt no. http://www.uniprot.org/uniprot/P20851, aa: 137–252) were cloned as previously described (Dervillez *et al.*, 2006). FHR4 was spanned between Bgl2 and BspE1, (c‐myc tag, a 5× (SGGGGS) spacer (5L), C4bpα and a His tag, and the last four components were cloned between BspE1 and XbaI. The 2D3 or 47D5 V_H_H anti‐HER2 was cloned in the same expression cassette with His tag to generate bifunctional heteromultimers lacking FHR4 or with FLAG tag to generate trifunctional heteromultimers. eGFP TrF was spanned between Bgl2 and BspE1, a 1× (SGGGGS) spacer (1L) and SCR3.C4bpβ, the last two components cloned were between BspE1 and XbaI.

### 2.3 Production of multifunctional heteromultimers

During Step 1, HEK293 cells were cotransfected by using lipofectamine 2000 (Invitrogen, Thermo Fisher Scientific BVBA) with TrF and targeting function (TaF) expression vectors. The TaF vector contains either the 2D3 V_H_H anti‐HER2 (recognising trastuzumab‐competing epitope) or the 47D5 V_H_H anti‐HER2 (recognising pertuzumab‐competing epitope) genes, as summarised in Fig. S1. Stable transfection was established using puromycin (InVivogen, Toulouse, France). Bifunctional heteromultimer‐containing crude supernatants of single‐isolated clones were tested on BT474 breast cells by flow cytometry using an anti‐FLAG or an anti‐His antibody. Single clones expressing the highest amounts of bifunctional heteromultimers were expanded. During Step 2 (Fig. S1), bifunctional heteromultimer‐expressing selected clones were cotransfected with AP‐effector function (EFF) vectors containing FHR4 and pTK‐Hygro (Clontech Laboratories Inc., Takara Bio Europe, Saint‐German‐en‐Laye, France). Stable transfected cells were selected using both puromycin and hygromycin (Carl Roth GmbH Co. KG, Karlsruhe, Germany). Supernatants from single‐isolated clones were screened for the mean FHR4 expression on BT474 target cells. Ratios of mean fluorescence intensity (MFI) His of bifunctional heteromultimers with FLAG and with His correspond, respectively, to lowest (His = 0) and highest (His = 7) FHR4 valence to eGFP MFI and allow to establish a calibration curve. His/eGFP MFI ratios from each single‐isolated clone were further assigned to the calibration curve to determine the mean FHR4 valence.

### 2.4 Multimer purification by using His‐Trap affinity chromatography

Multimer‐expressing HEK293 cells were cultured in FBS‐free Opti‐MEM medium (Life Technologies Europe BV). Supernatants were purified using BioLogic DuoFlow 10 medium‐pressure liquid chromatography system (FPLC; Bio‐Rad Laboratories NV) connected to Nickel His‐Trap 5 mL columns (GE Healthcare, VWR, Leuven, Belgium). Elution was performed using 20 mm phosphate buffer, pH 7.4 that contained 500 mm of sodium chloride (NaCl) and 500 mm of imidazole (Sigma‐Aldrich). Purified multimers were concentrated on Amicon Ultra 15, 50 K MWCO (Millipore‐Merck Chemicals NV/SA, Evere, Belgium), dialysed against PBS using Slide‐A‐Lyzer dialysis cassettes with 20K MWCO (Thermo Fisher Scientific) and quantified using NanoDrop 1000 (Thermo Fisher Scientific). To further enrich in high‐FHR4 valence molecular species, trifunctional FHR4‐heteromultimers were purified using an improved protocol including a first washing step of 50 mm imidazole followed by 125 mm imidazole to partly eliminate the molecular species with low‐FHR4 valence. A final two‐step elution was then performed using 1 m imidazole introducing a 2 h‐stop/resume in‐between (Fig. S3). Collected fractions from steps with 1 m imidazole peak 1 and peak 2 were pooled as fractions (f2) and 3 (f3), respectively. The fractions were concentrated, dialysed and quantified as described above in this section.

### 2.5 Multimer‐mediated AP activation, MAC binding and CDC

To assess multimer‐directed C3b deposition on HER2‐expressing tumour cells, purified FHR4/V_H_H(T), FHR4/V_H_H(P) multimers and control therapeutic antibodies were incubated separately or in combinations at saturating concentrations (15 µg·mL^−1^) with SK‐OV‐3, BT474 or SK‐BR3 tumour cells in gelatin veronal buffer containing CaCl_2_ and MgCl_2_ (GVB^++^) and 30% ABO‐compatible normal human serum (NHS) from a healthy donor for 30 min at 37 °C. After washing with PBS/1% decomplemented FBS, cells were then stained with a mouse anti‐human C3b mAb and a rabbit anti‐His pAb, followed by anti‐mouse IgG/APC and anti‐rabbit IgG/PE secondary antibodies for flow cytometry, or a mouse anti‐human C5b‐9 antibody to measure the MAC binding on cells. For multimer‐mediated CDC, 15 µg·mL^−1^ of multimers (or antibodies) was incubated with SK‐OV‐3, BT474 or SK‐BR3 cells in GVB^++^ supplemented with 30% ABO‐compatible NHS for 30 min at 37°C. Cells were incubated with a live/dead (L/D) near IR and fixed with 1% paraformaldehyde (PFA) to provide the percentage of dead cells. Percentage of dead cells was expressed as number of dead cells (live/dead‐positive cells) divided by the total number of cells analysed. FH‐mediated dose–response inhibition of on C3b deposition induced by the optimised high‐FHR4 valence multimer was performed by incubating SK‐OV3 cells with constant concentrations of purified FHR4/V_H_H(T) multimers (15 µg·mL^−1^) together with twofold serial dilutions of commercial soluble FH (starting at 125 µg·mL^−1^) in GVB^++^ containing 30% ∆C5HS for 30 min at 37 °C.

To demonstrate that FHR4‐heteromultimers activate the AP, SK‐OV3 cells (10^5^ cells per condition) were incubated for 30 min at 4 °C with saturating concentrations (30 µg·mL^−1^) of either FHR4/V_H_H(T) + FHR4/V_H_H(P) multimers, trastuzumab + pertuzumab, the V_H_H(T) control multimer or no molecules. After washing with PBS/1%FBS, cells were then incubated with 30% NHS in (a) GVB^++^; or (b) gelatin veronal buffer containing MgCl_2_ and EGTA (without CaCl_2_; GVB^+^); (c) 30% ∆C1qHS; (d) 30% ∆FBHS; (e) 30% ∆FBHS + 200 µg·mL^−1^ commercial FB; or (f) with 30% ∆C5HS for 30 min at 37 °C. After one washing with PBS/1%FBS, cells were stained with either an anti‐human C3b (7C12) or an anti‐human C4d mAb for 30 min at 4 °C, followed by a staining with an anti‐human IgG antibody PE‐conjugated. After washing, cells were fixed with PBS/1% PFA and analysed using flow cytometry.

### 2.6 Confocal microscopy analysis

To visualise multimer‐mediated AP activation and MAC binding by confocal microscopy, BT474 cells were incubated with 20 µg·mL^−1^ purified optimised FHR4/V_H_H(T) multimers in GVB^++^ supplemented with 25% ABO‐compatible NHS or heat‐inactivated NHS (HIS). The cells were stained with mouse anti‐human C3b/iC3b (7C12) or C5b‐9 (aE11) mAb, followed by an anti‐mouse IgG AF546‐conjugated secondary antibody. Cells were then fixed with 1% of PFA. Images were captured at 40 or 60‐time magnifications using a Zeiss LSM510 confocal microscope (Zeiss International, Oberkochen, Germany). Images were recorded at different focal planes; Z stacks from ~ 22 stacked‐up sections were generated by incrementally stepping through the cell volume using a focal drive. The Z sections were stacked using image j (NIH, Bethesda, MA, USA) to reconstruct three‐dimensional (3‐D) images.

### 2.7 Surface plasmon resonance analysis

Surface plasmon resonance (SPR) experiments were conducted using a Biacore 3000 system (GE Healthcare Europe GmbH, Diegem, Belgium). CM5 sensor chips were used with HBS‐EP buffer (10 mm Hepes pH 7.4, 150 mm of NaCl, 3 mm of EDTA and 0.05% of surfactant P20; GE Healthcare). Activation of surfaces and ligand immobilisations were achieved with the primary amine coupling kit according to the manufacturer’s instructions. Ten thousand RU of each of the purified high‐FHR4 valence trifunctional heteromultimers [FHR4/V_H_H(T)] from the first (Clone 2E3) and the third (Clone IA3) single‐cell sortings, as well as from the FHR4‐free control bifunctional heteromultimers [V_H_H(T), clone B2] (10 µg·mL^−1^ in 10 mm of acetate buffer, pH 4.5), was immobilised onto the separate flow paths. The mock activated–deactivated reference channel was treated with the amine coupling kit without ligand. Increasing serial concentrations of C3b (2.5, 5, 10 and 20 µg·mL^−1^ in HBS‐EP buffer supplemented with 20 mm MgCl_2_) were successively injected into the different flow paths for 5 min at 30 µL·min^−1^, followed by 5‐min dissociation. The surfaces were regenerated after each cycle with a 15‐s pulse of SDS 0.05% at 20 µL·min^−1^. Kinetic curves were mock subtracted, then the means for each concentration curve, the zero concentration curve and the corresponding reference curve were subtracted. The curves were fit with a 1 : 1 Langmuir model to determine the corresponding k_A_ and k_D._ The kinetics analysis was conducted with the BIAevaluation software v4.1 (Biacore 3000; GE Healthcare).

### 2.8 SDS/PAGE, SYPRO Ruby protein gel staining and western blotting analysis

One microgram of each of the purified heteromultimers was diluted in Laemmli sample buffer (Bio‐Rad Laboratories) and analysed under nonreducing or reducing conditions (10% of β‐mercaptoethanol; Sigma‐Aldrich) using SDS/PAGE (4–15% gradient precast gels; Bio‐Rad Laboratories) followed by SYPRO Ruby protein gel staining according to the user’s manual (Thermo Fisher Scientific). The SYPRO Ruby‐stained gels were scanned using the Typhoon Trio imager (laser 488 nm, filter 610/BP30; GE Healthcare Laboratories) and the typhoon scanner control v5.0 software (Amersham/GE Healthcare, Chicago, IL, USA). For western blotting (WB), 50 ng of the proteins was analysed by SDS/PAGE under nonreduced or reduced conditions and then transferred to a 0.2‐µm polyvinylidene fluoride membrane (GE Healthcare). Membranes were blocked in 5% nonfat dried milk in PBS at room temperature for 30 min. Membranes were probed overnight at 4 °C with primary antibodies in the same blocking buffer. Membranes were washed with PBS containing 0.01% Tween (PBS‐T) and probed with the secondary antibody (when the primary antibody was not already conjugated to HRP in PBS‐5% milk at room temperature for 1 h). Membranes were washed three times in PBS‐T for 10 min before being revealed with the enhanced chemiluminescence solution (Western Lightning Ultra Chemiluminescent Kit, Perkin Elmer, Zaventem, Belgium). Chemiluminescence signals were captured using an ecl chemocam phospho imager and the chemostar imager software (both from Intas Science Imaging Instruments GmbH, Göttingen, Germany).

### 2.9 Multimer‐mediated complement‐dependent cellular phagocytosis

Peripheral blood mononuclear cells (PBMCs) from healthy donors (Luxembourg Red Cross) were purified using LymphoPrep solution (Elitech Group BV, Zottegem, Belgium) with density gradient centrifugation at 800 ***g*** for 30 min. PBMCs were incubated for 2 h in complete RPMI medium. Adherent cells were washed with Dulbecco’s PBS at 37 °C and incubated for 14 days at 37 °C with 5% CO_2_ in complete RPMI medium supplemented with M‐CSF (50 ng·mL^−1^). M‐CSF was replaced every 3 days. Monocyte‐derived M2 macrophages were trypsinised and plated into Lab‐Tek 8‐chamber slides (Nunc; Thermo Fisher Scientific). Forty‐eight hours later, macrophages were stained with PKH26 (Sigma‐Aldrich) according to the manufacturer’s instructions. BT474 target cells were stained with CFSE (Invitrogen, ThermoFisher) and then incubated for 30 min with 20 µg·mL^−1^ of (a) combined FHR4/V_H_H(T) and FHR4/V_H_H(P) multimers; (b) combined trastuzumab and pertuzumab mAbs; or (c) without multimers nor mAbs in GVB^++^ supplemented with 25% ∆C5HS, heat decomplemented or not. CFSE‐stained complement‐coated tumour cells were then washed and added to the KHP26‐stained M2 macrophages (ratio of BT474 to M2 was 2 : 1) and incubated for 18 h at 37 °C/5% CO_2_ in complete RPMI medium. Cells were fixed with 1% PFA, stained with DAPI and analysed with confocal microscopy as described in section 2.7. Three series of 10 pictures were taken for each experimental condition. Phagocytosis was evaluated on 400 macrophages for each condition by counting the percentage of phagocytic macrophages 22 h after the beginning of the phagocytosis assay. Statistical analyses were performed using graphpad prism 5.0 (GraphPad Software, San Diego, CA, USA), applying analysis of variance (ANOVA) tests and followed by Dunnett’s multiple comparison test. Significance was accepted when *P* values were < 0.05.

### 2.10 Analysis of the solution‐phase complement activation

To analyse whether the FHR4‐heteromultimeric immunoconjugates consume complement in the solution phase, a CH_50_ test was performed to measure the 50% haemolytic complement activity of serum (Costabile, 2010). Sheep red blood cells [50 µL red blood cell (RBC)·mL^−1^ PBS or 2.8·10^8^ RBC·mL^−1^] were sensitised with a rabbit haemolytic anti‐sheep erythrocyte serum (dilution 1/15 000) for 30 min at 4 °C. A mix of 90% NHS diluted in 10% GVB^++^ (90% NHS/10% GVB^++^) was used as source of complement in the CH_50_ assay. The relationship between the amount of complement used and the amount of RBC lysed is nonlinear but follows a sigmoidal curve. Thus, for precise measurements of the haemolytic activity of complement, a titration of 90%NHS/10%GVB^++^ dilutions in PBS was established and an endpoint of optimal serum dilution was chosen in the upper linear part of the curve, before the plateau is formed to avoid working in excess of complement. In our experimental design, a dilution of 1/50 of the 90%NHS/10%GVB^++^ serum corresponding to 75% haemolysis meets the optimal conditions. Then, twofold serial dilutions (starting concentration being 0.8 g·L^−1^) of FHR4/V_H_H(T), FHR4/V_H_H(P), V_H_H(T) heteromultimeric immunoconjugates, trastuzumab, pertuzumab antibodies used individually or in combination were incubated for 1 h at 37°C in 90% NHS/10% GVB^++^. A serial dilution of aggregated IgG was prepared in parallel as positive control for phase solution complement consumption. The blank and tubes for spontaneous and total lysis were prepared with SSE alone, or SSE diluted in PBS or water, respectively. The different sera were diluted 1/50 in PBS and were incubated with SSE for 30 min at 37 °C. After centrifugation, the supernatants were collected and the absorbance of the samples was read at 416 nm. The % lysis for each sample was calculated using the following formula: [(OD_416_ (sample) − OD_416_ (Blank))/(OD_416_ (total lysis) − OD_416_ (Blank))] × 100.

## 3 Results

### 3.1 Structure–function relationship between FHR4 valence and AP activation on HER2‐overexpressing tumour cells

**Figure 1:**
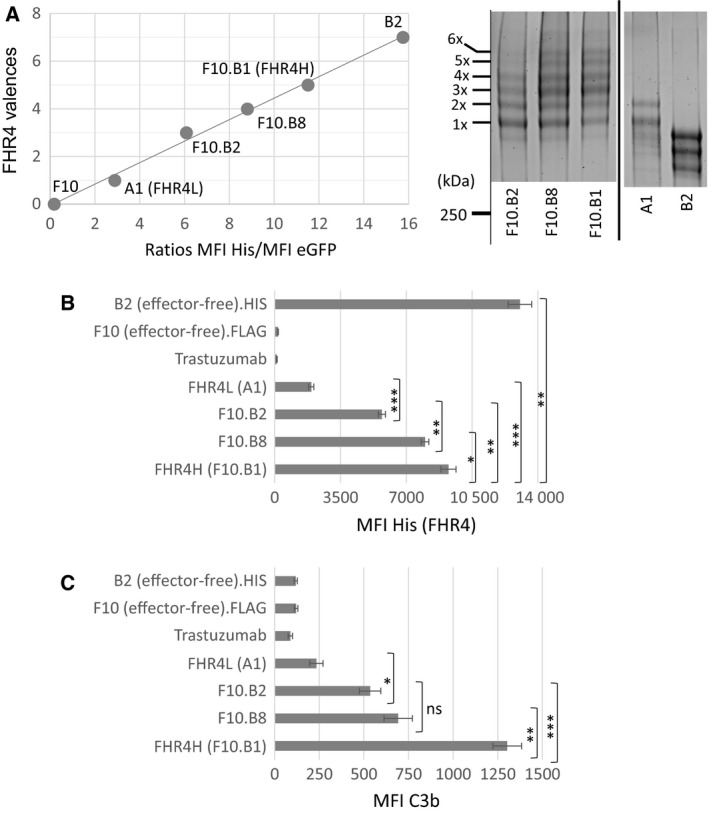
The mean FHR4 valence on HER2 cells correlates with AP activation levels and subsequent amounts of C3b deposition. (A) The mean FHR4 valence of four FHR4/V_H_H(T) trifunctional heteromultimer‐containing supernatants from four different cell clones was analysed by flow cytometry using SK‐OV3 cells and His and eGFP staining. Supernatants containing V_H_H(T).FLAG (clone F10) or V_H_H(T).His (clone B2) control bifunctional heteromultimers – lacking FHR4 – and displaying 0‐time (lowest) and seven‐time (highest) His, respectively, were used to establish a calibration curve according to their His MFI/eGFP MFI ratios. Since the FHR4 complement effector‐ and the V_H_H anti‐HER2 TaFs are coexpressed within the trifunctional heteromultimers, the global valence of both functions is seven. FHR4 valence within the multimers varies thus from 1 (and 6xV_H_H) to 6 (and 1xV_H_H). The third eGFP TrF is a single molecule within the multimers, and the His tag is on the same arm as FHR4 (but not the V_H_H), thus the His MFI/eGFP MFI ratio represents the mean FHR4 densities on SK‐OV3 cells. For each clone, the His MFI/eGFP MFI ratio is reported on the calibration curve, allowing evaluation of the mean FHR4 valence on the *y*‐axis. Note that the multimers in the supernatants are at saturating concentrations. On the right, SDS/PAGE and SYPRO Ruby protein gel staining depicts the molecular species from 1x to 5xFHR4 and showed that clone F10.B1 displayed molecular species with higher FHR4‐valences as compared to A1 or F10.B2. The bold line separates lanes from different gels. (B, C) FHR4 multimer‐mediated C3b deposition correlates with FHR4 densities on BT474 cells. His MFI of each clone and controls was measured (B) and was compared to the levels of C3b deposition (C). For F10.B8 and F10.B1, a FHR4 increase by factor 1.15 leads to a C3b increase by factor 1.9. Data are presented as mean values ± SD of *n* = 3 independent experiments. Statistical analysis was performed using a two‐tailed paired *t*‐test. *P < 0.05, **P < 0.01 and ***P < 0.001.

Directing local complement activation towards undesired cells (e.g., tumour cells) is an attractive, but challenging, approach (Carter and Lieber, 2014; Diebolder *et al.*, 2014). We hypothesised that multimerisation of AP effectors at the surface of target cells using the C4bp C scaffold could achieve this goal. We first produced a trifunctional heteromultimer displaying (a) FHR4; (b) the 2D3 anti‐HER2 V_H_H recognising a trastuzumab‐competing epitope [FHR4/V_H_H(T)] (Patent US12744991); and (c) eGFP (Fig. S1). Bifunctional heteromultimers [V_H_H(T)] were first produced (Fig. S1) with either a FLAG tag (Clone F10) or a His tag (Clone B2). Clone F10 was further transfected with the FHR4 construct to generate trifunctional heteromultimers. His/eGFP MFI ratios of four representative clones with different FHR4 valences are depicted in Fig. 1A and spanned between the His/eGFP MFI ratios of the bifunctional heteromultimers, Clones F10 (0.17) and B2 (15.75), correspond, respectively, to a mean 0xHis and 7xHis on the *y*‐axis of the calibration curve (Fig. 1A, left). A SYPRO Ruby protein gel staining analysis (Fig. 1A, right) confirmed that multimers with higher FHR4 valences displayed molecular species with higher molecular weight (MW) because of the higher content of FHR4 (120 kDa). The level of C3b deposition correlated with mean FHR4 valences within each multimeric clone (Fig. 1B,C). Clones B2 and F10 negative controls (both lacking FHR4) or trastuzumab did not elicit C3b activation on BT474 tumour cells expressing HER2 (Fig. 1C). The low valence FHR4L (Clone A1) showed a weak C3b activation (234 MFI), whereas high valence FHR4H‐mediated C3b deposition was more than 10 times higher than clones B2 or F10 and 5.6 times higher than FHR4L (1303 vs 234 MFI, respectively). FHR4 stoichiometry within the multimeric immunoconjugates is therefore the key element to modulate FH‐mediated C3‐convertase decay and FHR4‐induced AP activation.

When comparing the ‘high‐FHR4 valence group’ that include clones F10.B1/FHR4H & F10.B8 to the ‘low‐FHR4 valence group’ that include clones F10.B2 & A1/FHR4L, a respective increase of mean FHR4 densities of 1.15 (FHR4 MFI ratio F10.B1/F10.B8 = 9246/8000) and 2.93 (FHR4 MFI ratio F10.B2/A1 = 5700/1947) led to an increase of C3b deposition of 1.88 (C3b ratio F10.B1/F10.B8 = 1303/692) and 2.28 (C3b ratio F10.B2/A1 = 535/234), respectively. These results of FHR4 MFI ratios for the high‐ and low‐FHR4 valence groups were divided by the C3b MFI ratios of the same groups, respectively (1.15/1.88 and 2.93/2.28, respectively). This ratio is inversely proportional to the efficacy of C3b deposition relative to the increase of mean FHR4 densities. The ratio was 0.61 and 1.285 for the high and low‐FHR4 valence groups, respectively. This indicates that AP activation is twice more efficient in the high‐FHR4 valence group than in the low‐FHR4 group and twice as much FHR4 density increase is required in a first phase of AP activation as compared to the later phase – when AP activation is already engaged – to lead to an equivalent C3b deposition efficacy.

A similar approach was used to generate a trifunctional heteromultimer displaying the 47D5 anti‐HER2 V_H_H recognising a pertuzumab‐competing epitope [FHR4/V_H_H(P)] (Patent US12744991).

### 3.2 Selection of high‐FHR4 valence immunoconjugates using sequential single‐clone sorting

**Figure 2:**
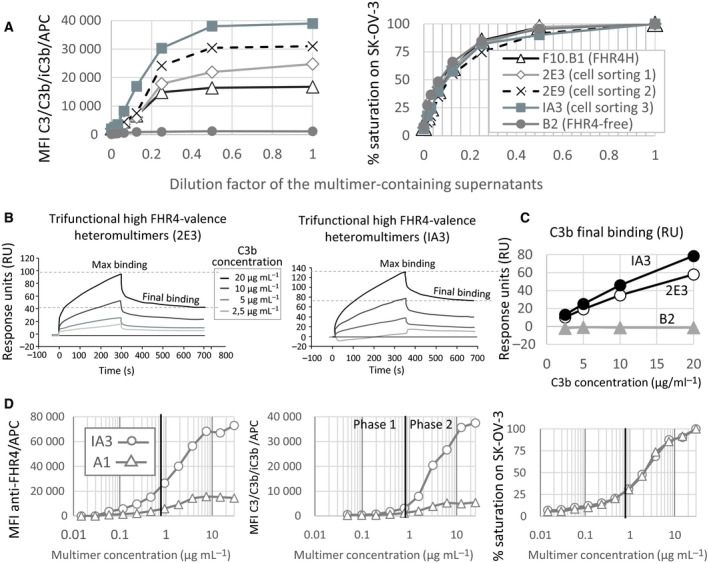
Sequential single‐cell sorting allowed the selection of cell clones expressing multimers with enhanced FHR4 mean valence and improved AP activation efficacy. (A) FHR4/V_H_H(T) trifunctional heteromultimer‐containing supernatants from clones F10.B1 (FHR4H) and those after three sequential cell sortings (2E3, 2E9 and IA3, respectively) showed increased FHR4 mean valence which correlates with an increase of MFI for C3b of a factor 2.32 between FHR4H and IA3. (B) Surface plasmon resonance analysis showed higher C3b binding specificity for FHR4 between the 1st (clone 2E3) and the 3rd (clone IA3) cell sorting. Sensorgrams for 2E3 (left) and IA3 (right) are depicted. (C) C3b binding increased of a factor 1.35 between captured 2E3 and IA3 multimers (58 and 78 RU for 2E3 and IA3, respectively) was observed which correlates with increase mean FHR4 valence within multimers. (D) C3b activation takes place according to a bimodal reaction only for the high FHR4 IA3 clone but not the low‐FHR4 A1 clone. At same multimer densities on SK‐OV‐3 cells (right graph), only FHR4 densities from clone IA3 – but not from clone A1 – (left graph) succeeded in overcoming the CIT (middle graph), the critical point where the competition of FHR4 with FH for C3b is strong enough to inhibit FH and to subsequently engage efficient AP‐positive amplification loop, leading to a linear increase of C3b deposition (phase 2 in the middle graph).

We further assumed that clonal selection might strongly influence the multimer expression pattern and should enhance the mean FHR4 valence within FHR4/V_H_H(T) trifunctional heteromultimers, especially when the intracellular expression of monomeric EFF of a selected clone is initially higher than that of the monomeric TaF. Clone F10.B1 (FHR4H) displaying high‐FHR4 valence multimers was sequentially submitted to three single‐cell sortings. Clones 2E3, 2E9 and IA3 were issued from the first, second and third single‐cell sortings, respectively (Fig. S2). C3b activation on SK‐OV3 cells increased by factors of 1.48 for Clone 2E3, 1.85 for Clone 2E9 and 2.32 for Clone IA3 at saturating multimer concentrations (240 nm) when compared with the original F10.B1 clone (Fig. 2A). SPR was used to confirm enhanced C3b binding capacity of purified FHR4‐multimers from clone IA3 (issued from the third single‐cell sorting) when compared to clone 2E3 (issued from first single‐cell sorting; Fig. 2B). We observed no C3b binding either on the FHR4‐free B2 control multimer‐coated surface or on reference surface without multimer (data not shown). At 20 µg·mL^−1^ of injected C3b, the maximum C3b binding for Clone IA3 was higher than for Clone 2E3 (136 vs 100 RU, respectively), and the final C3b binding was similarly enhanced by a factor of 1.36 (78.6 vs 58 RU, respectively; Fig. 2C). The calculated equilibrium association constants (*K_A_*) were 3.65 × 10^6^ m ^−1^ for Clone 2E3 and 4.48 × 10^6^ m ^−1^ for Clone IA3. The equilibrium dissociation constants (*K*_D_) were 2.74 × 10^7^ m for Clone 2E3 and 2.23 × 10^7^ m for Clone IA3, respectively, showing a similarly high affinity of the immunoconjugates for C3b. The dose–response relationship between FHR4 density and AP‐activation efficacy was compared between the optimised high‐FHR4 valence (IA3) and the initial low‐FHR4 valence (A1) heteromultimers (Fig. 2D). At saturating and similar concentrations of each multimer (Fig. 2D, right), FHR4 densities were five times higher for IA3 (72 953 MFI) than for A1 (14 487 MFI; Fig. 2D, left). Subsequent C3b activation was seven times higher for IA3 (37 541 MFI) than for A1 (5432 MFI) multimers (Fig. 2D, middle). A detailed analysis of the dose–response of multimer‐mediated AP activation highlighted that the IA3 concentration of 0.8 µg·mL^−1^, corresponding to an MFI of 26 496 for FHR4 (Fig. 2D, left), represents the critical FHR4 density to enable efficient AP activation (Fig. 2D, middle). Further linear increase beyond this critical density, and up to 20 µg·mL^−1^, coincides with a linear increase of C3b deposit, inflecting from 7.5 µg·mL^−1^ IA3 multimers, and establishing a plateau with a final MFI of 72 953 for FHR4. In contrast, for A1 multimers, FHR4 densities reached a plateau from a starting concentration of 5 µg·mL^−1^, with a maximal MFI of 14 487, representing only 55% of the FHR4 density required to start activating AP. High FHR4 valence multimer‐mediated C3b activation appeared to occur on target cells according to a bimodal reaction. The first phase takes place below the complement inhibitory threshold (CIT; Fig. 2D, middle) when FHR4 density cannot fully compete with FH. The second phase corresponds to the phase above the CIT, where FHR4 densities are sufficient to counteract FH‐controlled complement inhibition.

**Figure 3:**
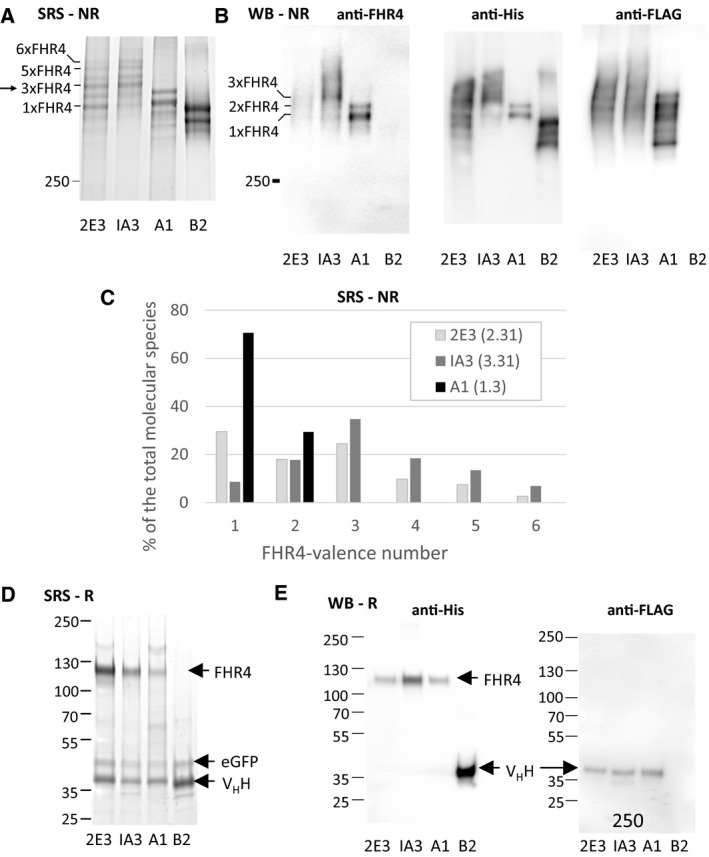
Analysis of the molecular patterns of purified A1, 2E3 and IA3 multimers using SRS or WB with anti‐FHR4, anti‐His or anti‐Flag antibodies under nonreducing conditions (NR, A, B, C) or under reducing conditions (R, D, E). (A, B) IA3 multimers display molecular species with higher FHR4 valences than their 2E3 counterparts. A1 low‐FHR4 valence multimers display two main bands that correspond to 1x and 2xFHR4 molecular species. B2 control multimers display two lower bands, both lacking FHR4, and corresponding to the 7α1β and 6α1β molecular species, with the V_H_H anti‐HER2 and eGFP functions being fused to C4bpα and C4bpβ, respectively. A lower third band, much less represented (~ 6% of the total), could correspond to a multimeric molecular species lacking eGFP function. (C) The percentages of each molecular species within the 2E3, IA3 and A1 multimers are shown by using the method of the calculation of the area under the curve. (D) SRS under reducing conditions showed each function as a monomer: FHR4, V_H_H anti‐Her2 and eGFP of 120, 40 and 50 kDa molecular weight, respectively. The band corresponding to FHR4 is absent for clone B2 which lacks FHR4 function. (E) WB under reducing conditions. Using the anti‐His Ab, FHR4.C4bpα.His8x function was illuminated for 2E3, IA3 and A1, but not B2 which lacks FHR4 (e left). Instead, the 2D3 V_H_H.C4bpα.His function was revealed in B2 (e left, lane 4). At same protein concentration, the band was stronger for IA3 – when compared to 2E3 and A1 – which harbours higher FHR4 valence. When using the anti‐FLAG Ab, the V_H_H anti‐HER2.C4bpα.FLAG function was revealed for 2E3, IA3 and A1, but not B2, which harbours a His tag.

We next used SDS/PAGE combined either with SYPRO Ruby protein gel staining or with WB to analyse the molecular pattern of the optimised immunoconjugates after clonal selection. Under nonreducing conditions, SYPRO Ruby staining (SRS) showed that the bifunctional Clone B2 (Fig. 3A, lane 4) displayed two main bands, corresponding to the molecular species 7xV_H_H/1x eGFP and 6xV_H_H/1x eGFP. A third lower minor band may correspond to a hexameric form lacking the eGFP β chain. The A1 multimer molecular pattern (Fig. 3A, lane 3) displayed two main bands that correspond to molecular species with 1x and 2xFHR4, respectively. 2E3 multimers (Fig. 3A, lane 1) displayed six bands that correspond to molecular species with 1x to 6xFHR4 valences, including two main bands for 1xFHR4 and 3xFHR4 valence molecular species. When compared with 2E3, IA3 multimers (Fig. 3A, lane 2) displayed: (a) more molecular species with 4x, 5x and 6xFHR4 valences; and (b less molecular species with 1x and 2xFHR4 valence. A WB analysis under nonreducing conditions (Fig. 3B) confirmed the molecular patterns depicted by the SYPRO Ruby protein gel staining analysis when revealed with either the anti‐His or the anti‐FHR4 antibody (Fig. 3B). The analysis showed 3xFHR4 to 6xFHR4 molecular species for IA3 (Fig. 3B, lane 2) that were absent in 2E3 (Fig. 3B, anti‐His, lane 1). A FLAG analysis showed a stronger intensity for A1 than for 2E3 or IA3, in agreement with higher V_H_H anti‐HER2 valence in A1 (Fig. 3E). The proportion of each molecular species was determined by measuring the area under the curve for each peak of the different clones detected by the SYPRO Ruby protein gel staining analysis (Fig. 3C). Overall, a clear shift towards molecular species with higher FHR4 valence for IA3 was revealed, while a shift towards lower FHR4 valences was observed for A1. A 2.5 times higher FHR4 valence for IA3 than for the A1 multimers was observed, as well as a 1.4 times higher FHR4 content in IA3 than in 2E3, in accordance with C3b binding (factor 1.35, Fig. 2C) and C3b deposition (factor 1.6, Fig. 2A) enhancements. The SRS analysis under reducing conditions (Fig. 3D) showed three bands that correspond to the monomeric forms of FHR4.C4bpα.His8x, the eGFP.SCR3.C4bp β and the V_H_H anti‐HER2.C4bpα.FLAG, respectively, for each clone. WB analysis under reducing conditions (Fig. 3E) revealed stronger intensity of FHR4 (His antibody) for Clone IA3 as compared with Clones 2E3 and A1 (Fig. 3E, left, lane 2) and a similar expression of the V_H_H anti‐HER2 (FLAG antibody, Fig. 3E).

### 3.3 Optimised FHR4 immunoconjugates with enriched molecular species promote enhanced C3b depositions, MAC binding and CDC

**Figure 4:**
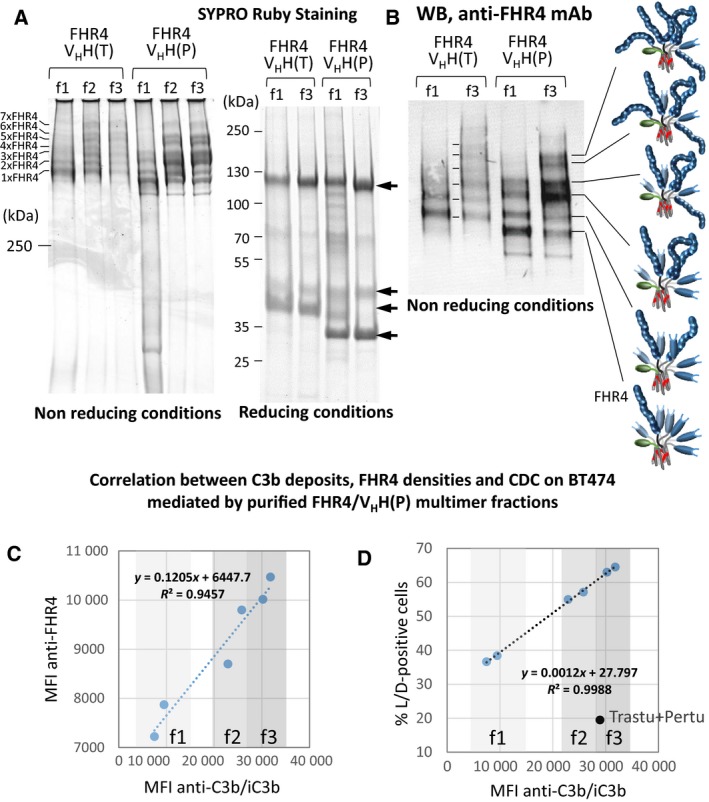
FHR4/V_H_H(T) or FHR4/V_H_H(P) multimer‐containing cell culture supernatants were purified using His‐Trap and FPLC chromatography and a three‐step elution was then performed. The first step using 120 mm imidazole, the 2nd and 3rd steps using 1 m imidazole with an at least 2 h‐stop in‐between the last two steps. Pooled collected fractions from each step are called f1, f2 and f3, respectively. (A) SRS analysis of f1–f3 fractions of purified FHR4/V_H_H(T) or FHR4/V_H_H(P) multimers under nonreducing (NR; left) and reducing (R; right) conditions. f1 contains mainly low‐FHR4 valence molecular species (1x and 2x FHR4), as well as contaminants. f2 and f3 display relative similar patterns, with higher FHR4‐valence molecular species, notably 3x–6xFHR4. Under reducing conditions, top‐down reduced bands (arrows) correspond to FHR4.C4bpα.His, eGFP.C4bpβ, V_H_H(T).C4bpα.FLAG and V_H_H(P).C4bpα.FLAG monomers, displaying apparent MW 120, 50, 40 and 33 kDa, respectively. (B) WB analysis under NR of f1 and f3 from FHR4/V_H_H(T) or FHR4/V_H_H(P) multimers using a mouse anti‐human FHR4 mAb confirmed the SRS analysis; f1 fractions contain lower FHR4‐valence molecular species. (C, D) Correlation between C3b deposition, mean FHR4 densities and CDC on BT474 cells mediated by purified FHR4/V_H_H(P) multimer fractions. BT474 cells were incubated with saturating concentrations of f1 to f3 (20 µg·mL^−1^) from two different FHR4/V_H_H(P) purifications, then with 30% NHS at 37 °C. Cells were analysed by flow cytometry for mean FHR4 densities, C3b deposition and CDC. (C) Correlation between mean FHR4 densities and C3b deposition. (D) Correlation between the percentage of dead cells (i.e., live/dead‐positive cells) and C3b deposition. The black dot represents the comparison with the use of combined trastuzumab and pertuzumab, showing the same range of C3b deposition as f3, but a far weaker cell death.

We performed a His‐Trap purification of the FHR4‐high valence trifunctional heteromultimers with either trastuzumab‐competing [FHR4/V_H_H(T)] or pertuzumab‐competing [FHR4/V_H_H(P)] anchoring moieties using a 3‐step sequential elution (Fig. S3) to further enrich in molecular species with higher FHR4‐valence. The SYPRO Ruby protein gel staining and WB analyses showed that low‐FHR4 valence multimers, as well as irrelevant contaminants, were in the f1 fractions (Fig. 4A left), while f2 and f3 displayed increasingly enriched molecular species with higher‐FHR4 valence. The analysis of mean FHR4 valence, AP activation and CDC on BT474 cells of f1 to f3 fractions of FHR4/V_H_H(P) His‐Trap purifications (Fig. 4C) showed a strong linear correlation between mean FHR4 valence and C3b deposition (r^2^ = 0.94) and between C3b deposition vs CDC (*r* ^2^ = 0.99; Fig. 4D). An increase of mean FHR4 valence of a factor 1.33 from f1 to f3 fractions led to an increase in deposited C3b by a factor of 3.8, consequently leading to an increase of cell death from 38% to 65% (factor 1.7; Fig. 4B,D). Since the difference of multimer‐induced CDC is narrow, for each FHR4/V_H_H(T) and FHR4/V_H_H(P) multimers, f2 and f3 fractions were pooled for further analyses and represented the lead FHR4/V_H_H(T) and FHR4/V_H_H(P) multimers that were used for the further experiments.

**Figure 5:**
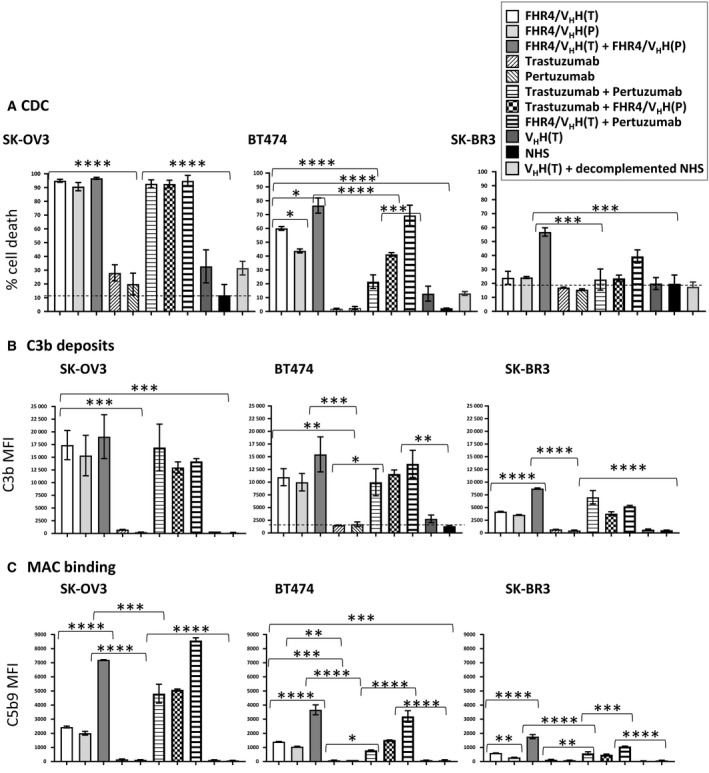
Multimer/mAb‐mediated CDC (A), C3b deposition (B) and MAC formation (C) on SK‐OV3, BT474 and SK‐BR3 tumour cell lines. Optimised FHR4/V_H_H(T), FHR4/V_H_H(P) trifunctional heteromultimers, control V_H_H(T) bifunctional heteromultimer, and trastuzumab and pertuzumab were tested individually or in combination at saturating concentrations (20 µg·mL^−1^), and incubated with 30% NHS at 37 °C for 30 min. Negative controls are control multimers [V_H_H(T)] and NHS alone. (A) Cells were stained with live/dead. Percentages of dead cells (live/dead‐positive cells) were analysed using flow cytometry. (B) Cells were stained with an anti‐C3b mAb (clone 7C12) followed by an anti‐mouse IgG PE‐conjugated pAb. (C) Cells were stained with an anti‐C5b‐9 mAb (clone aE11) followed by an anti‐mouse IgG PE‐conjugated pAb. Data are presented as mean values ± SD of *n* = 3 independent experiments; *P < 0.05, **P < 0.01, ***P < 0.001, ****P < 0.0001.

We further tested FHR4/V_H_H(T) and FHR4/V_H_H(P) multimeric immunoconjugates either individually or in combinations in three different cell lines expressing HER2 (SK‐OV3, BT474 and SK‐BR3 cells) for CDC (Fig. 5A), C3b deposition (Fig. 5B) and MAC binding (Fig. 5C). In SK‐OV3 cells, trastuzumab‐ and pertuzumab‐mediated CDC was poor when used individually (20–27% lysis), whereas 93% of cell death was achieved when both antibodies were combined. Most of SK‐OV3 cells were lysed by FHR4/V_H_H(T) (95%) or FHR4/V_H_H(P) (91%), reaching lysis of 97% when combined. For BT474 cells, cell lysis was 60%, 40% and 77% for FHR4/V_H_H(T), FHR4/V_H_H(P) and their combination, respectively, as compared to 21% lysis when using combined trastuzumab and pertuzumab. The combination of FHR4/V_H_H(T) with pertuzumab led to a CDC close to that of combined FHR4/V_H_H(T) + FHR4/V_H_H(P) heteromultimers (69% vs 76% cells death). In contrast, the other combination between trastuzumab and the FHR4/V_H_H(P) was significantly less efficient (*P* = 0.0006). For SK‐BR3 cells, only the combination of FHR4/V_H_H(T) and FHR4/V_H_H(P) led to a cell lysis of 57% and the combination of FHR4/V_H_H(T) and pertuzumab led to 39% cell lysis. Of note, the control multimer V_H_H(T) displayed some cell death (30%, 13% and 20% on SK‐OV3, BT474 and SK‐BR3, respectively) in a complement‐independent fashion, since cell death remained unchanged when using decomplemented NHS (data not shown).

In SK‐OV3 cells, levels of C3b deposition were highly correlated with CDC for all conditions used (Fig. 5A,B) but not with MAC binding, suggesting that a certain threshold amount of MAC densities was able to induce CDC in these cells. Regarding BT474 and SK‐BR3 cells, the efficacy of C3b deposition correlated well with CDC when the multimers were used alone but not when combined with mAbs (Fig. 5A,B): for the same levels of C3b deposition, CDC was about twice as low for combined mAbs than for FHR4 multimers. For BT474 cells, similar C3b MFI led to 20%, 60% and 43% cell lysis when using combined mAbs, FHR4/V_H_H(T) or FHR4/V_H_H(P), respectively. For SK‐BR3 cells, combined mAbs, or a combination of FHR4/V_H_H(T) and pertuzumab displayed C3b MFI of 7024 and 5265, whereas percentages of cell death were 23% and 40%, respectively. A clear correlation was observed between MAC binding and CDC for FHR4‐based multimers and FHR4‐based multimers in combination with mAbs in BT474 and SKBR3 cells as depicted in Fig. 5B,C, but not for combined mAbs. The two most favourable conditions for highest MAC binding and CDC in all three cell lines were the combination with the two FHR4‐based multimers and the combination of FHR4/V_H_H(T) with pertuzumab (Fig. 5B). For the three studied cell lines, combined FHR4‐multimers elicited more than twice MAC densities than when used individually. In contrast, mAbs used alone as well as the V_H_H(T) control multimer elicit strictly no MAC binding. This reinforces the fact the V_H_H(T) control multimer‐mediated cytotoxicity mainly observed for SK‐OV3 is not dependent upon complement activation.

Multimer‐mediated C3b deposition and CDC were further confirmed on BT474 cells using serial dilutions (Fig. S4A,B). C3b deposition and CDC were sustained at lower multimer concentrations when FHR4/V_H_H(T) and FHR4/V_H_H(P) multimers were combined than when they were used individually. Although the FHR4/V_H_H(T) and pertuzumab combination led to the highest C3b deposition and strongest CDC at the highest multimer concentration, the drop in the amounts of C3b deposition and related CDC were much faster than for combined FHR4/V_H_H(T) and FHR4/V_H_H(P) multimers, suggesting that pertuzumab reinforced the effect of FHR4/V_H_H(T) on AP activation and CDC only at highest concentrations.

**Figure 6:**
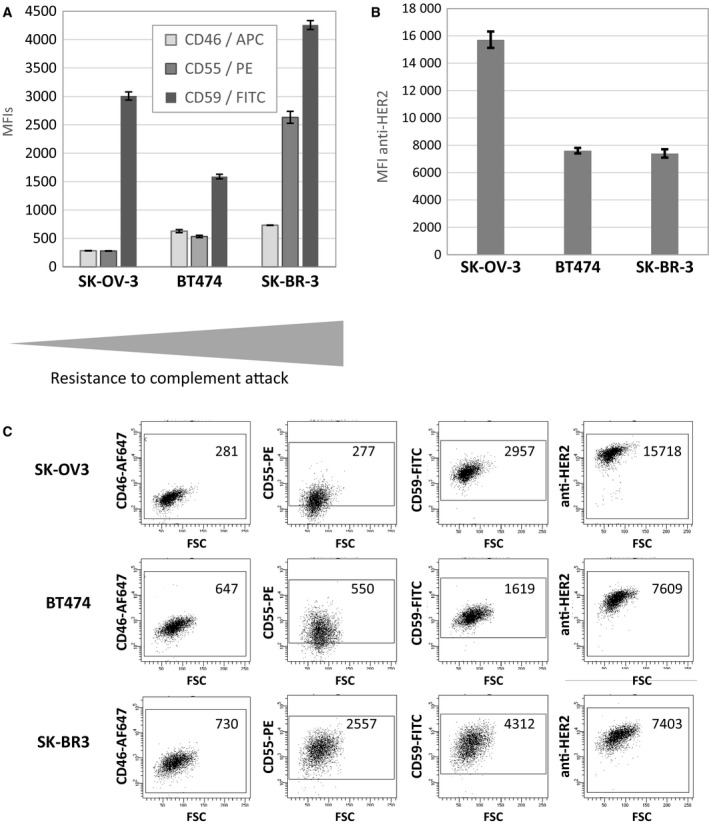
mCRP (A) and HER2 (B) relative densities in SK‐OV3, BT474 and SK‐BR3 tumour cell lines as determined by flow cytometry. (A) CD46‐APC, CD55‐PE and CD59‐FITC Abs were coincubated with the cells lines. (B) Trastuzumab was incubated at saturating concentrations with the cells and its binding was revealed with an APC‐conjugated anti‐human IgG. (C) Dot plot representation of CD46, CD55, CD59 and HER2 MFI on SK‐OV3, BT474 and SK‐BR3 HER2‐overexpressing tumour cell lines. Data are means ± SD of three experiments.

We next showed that the addition of FH lowered in a dose–response manner FHR4 enhancing effect on AP activation and subsequent C3b depositions on HER2‐overexpressing tumour cells (Fig. S4C). These data support that FH antagonised the C3b deposition mediated by the FHR4‐based immunoconjugates. Finally, to explain the diverse resistance to complement attack among the three different cell lines used, we measured the relative mCRP (Fig. 6A,C) and HER2 densities (Fig. 6B,C) on the three cell lines by flow cytometry. SK‐OV3 cells expressed about twice as much HER2 (displayed as MFI values) and lower CD46/CD55 densities compared to the two other cell lines, followed then by BT474 cells, in accordance with their increased resistance to complement attack as observed in section 3.3. SK‐BR3 cells were the most resistant cell line, displaying 5–10‐times more CD55 densities and 1.4–2.8‐times more CD59 densities than SK‐OV3 and BT474 cell lines.

To finally assess whether the FHR4‐heteromultimeric immunoconjugates activate the alternative (AP) vs the classical (CP) pathways, C3b and C4d deposition was measured in six different conditions (Fig. S5). In conditions 1 (NHS/GVB^++^), 5 (∆FBHS + FB) and 6 (∆C5HS), both AP and CP were functional, and C3b deposition was therefore observed when using combined FHR4‐multimers or combined mAbs, whereas C4d deposition was observed only when using combined mAbs and not FHR4‐multimers. In conditions 2 (NHS/GVB^+^) and 3 (∆C1qHS), only the AP was functional: the combined FHR4‐multimers induced C3b but no C4d deposition. In contrast, when the CP was only functional and not the AP in condition 4 (∆FBHS), only the combined mAbs induced C3b as well as C4d deposition and not the combined FHR4‐based immunoconjugates. When FB was added (condition 5), the C3b amplification loop (AP) was restored. Consequently, C3b deposition, but no C4d deposition was observed when using combined FHR4‐multimers (Fig. S5A,B).

### 3.4 Optimised FHR4‐immunoconjugates induce MAC binding on membrane and promote specific complement‐dependent macrophage‐mediated phagocytosis of BT474 target cells

**Figure 7:**
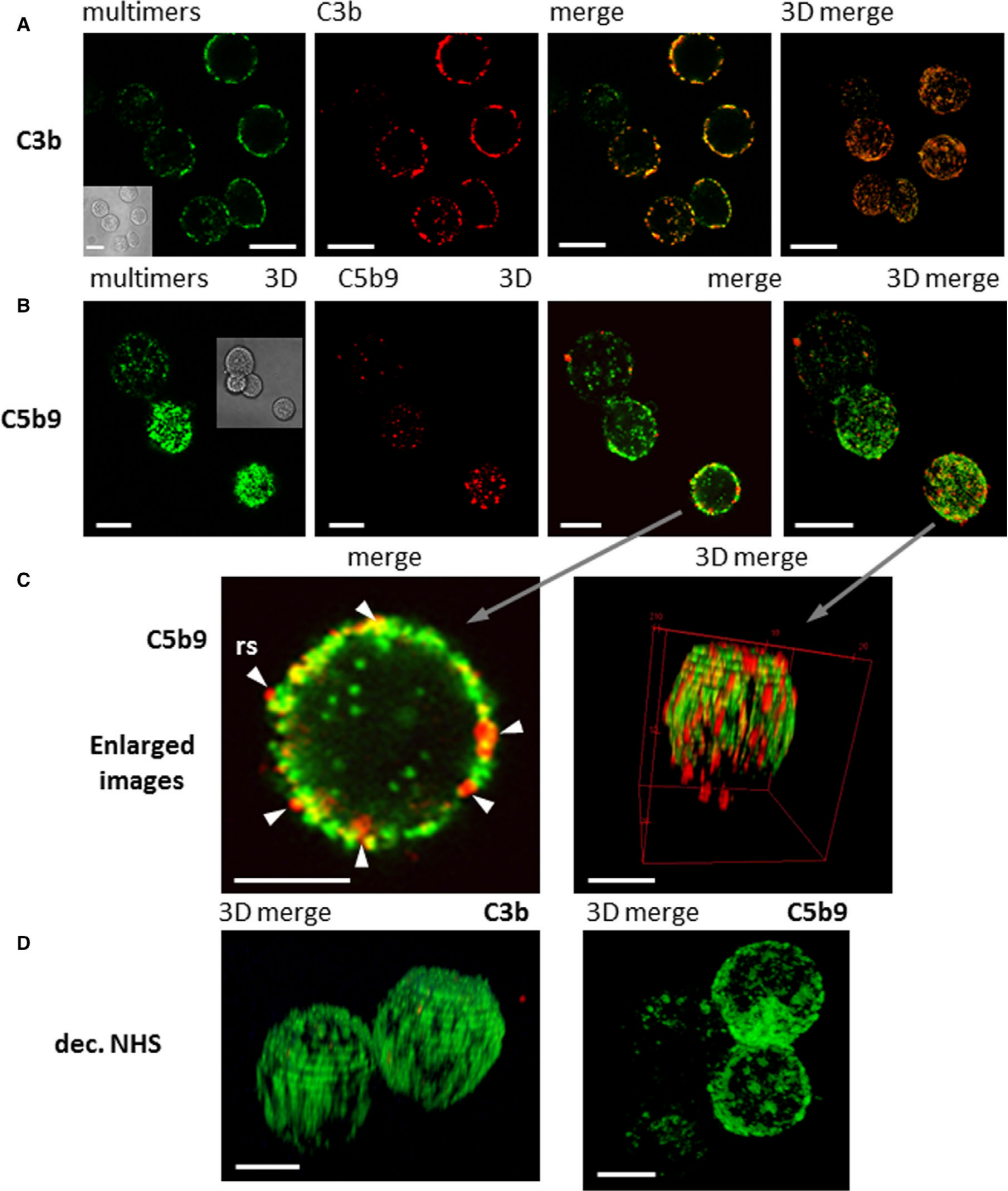
Confocal microscopy analysis of FHR4/V_H_H(T) multimer‐mediated C3b deposition and MAC formation on BT474 cells. (A) Colocalisation of C3b deposition and FHR4/V_H_H(T) multimeric immunoconjugate (clone IA3). IA3 multimers on the cell surface (green) and C3b staining (red) were merged as a one‐layer section. A strong colocalisation appears as yellowish‐orange staining. The far right image is a 3D merging that represents whole cells. The scale bars represent 10 µm. (B) MAC formation analysis. FHR4/V_H_H(T) Multimer (green) and C5b‐9 staining (red) were merged as one‐layer sections (third picture from left), or as a reconstruction of 3D stacked‐up one‐layer sections. Bright red spots were revealed with some colocalisation with the multimers as the yellow interface. The scale bars represent 10 µm. (C) Magnification of a single cell representing the details of inserted MAC. The magnification of a single section of a merged staining from (B), showing the C5b‐9 staining of a one‐layer section (left), or a 3D reconstruction of merged staining (right). The scale bars represent 5 µm. (D) 3D reconstruction of sections with merged multimer and C3b (left) or C5b‐9 staining (right) in conditions where decomplemented NHS was used showed no staining. The scale bars represent 5 µm.

We used confocal microscopy to visualise MAC binding and examine the colocalisation between the immunoconjugates and C3b or C5b‐9 on the surface of BT474 cells. FHR4/V_H_H(T) immunoconjugates (green) were relatively homogeneously distributed on the surface of the cells, displaying a strong colocalisation with the C3b deposition (red; Fig. 7A, third and fourth merged pictures) resulting in a yellowish‐orange colour. The C5b‐9 staining was, in contrast, much more dispersed and clustered than C3b staining with many bright red spots detected (Fig. 7B,C), indicating that MAC is mainly assembled further away from the multimers and at lower densities than C3b deposition. The magnification of a cell in Fig. 7C shows that the yellowish‐orange interface seems to span across the green staining and through the membrane. Bright red spots in Fig. 7C are visible either on top of or through a yellowish‐orange interface and sometimes make protrusions through the membrane and extend slightly towards the inside, which may represent MAC that are anchored through the membrane. When using decomplemented serum, we did not observe any C3b or C5b‐9 staining as depicted in Fig. 7D. Interestingly, we detected green multimer staining inside the cells in Fig. 7A–C. This intracellular staining indicated an endocytosis of HER2‐multimeric complexes, which are particularly visible when using the FHR4‐free B2 multimers harbouring 7xV_H_H anti‐HER2 valences (data not shown), which occurs in a complement‐independent manner.

**Figure 8:**
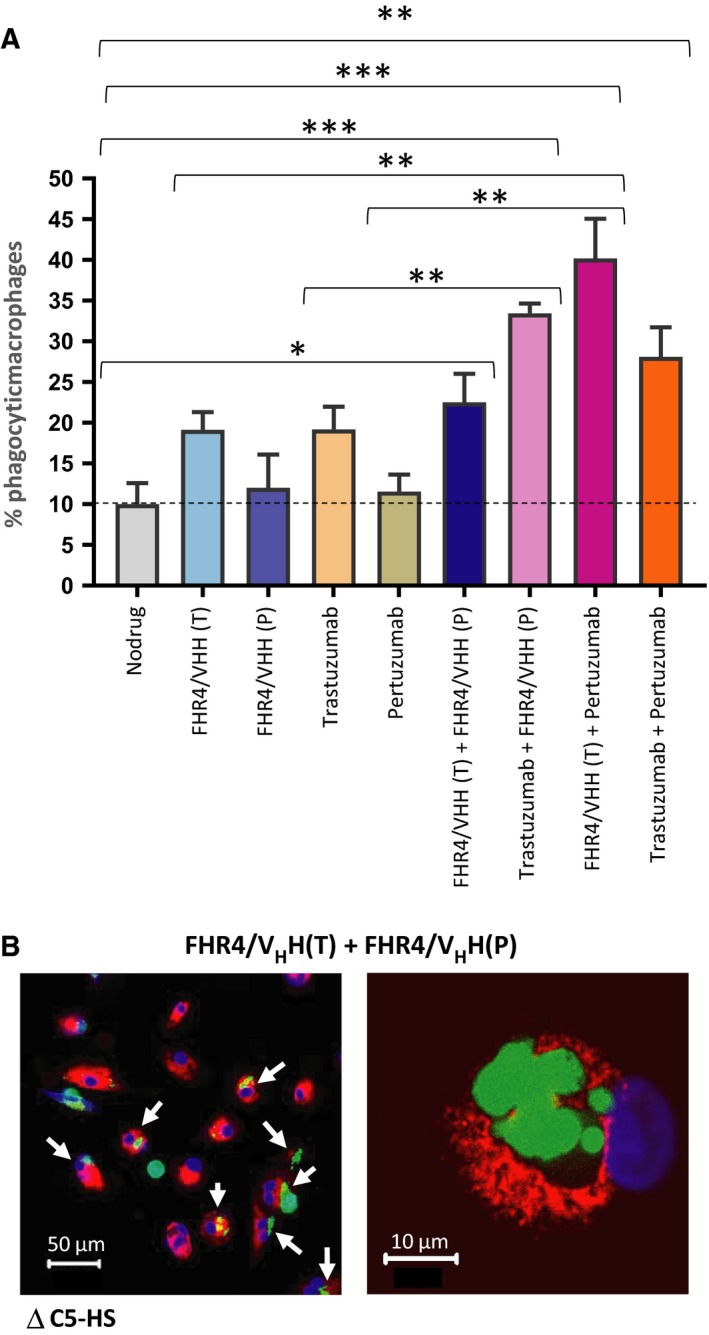
Complement‐dependent macrophage‐mediated phagocytosis of BT474 cells. (A) CFSE‐stained BT474 tumour cells were incubated with multimers and/or antibodies individually or in combination. Cells were incubated with 25% C5‐deficient human serum (∆C5HS) to prevent lysis. KPH26‐stained monocyte‐derived M2‐macrophages were then incubated with tumour cells (ratio 2 : 1) for 18 h. (A) Percentage of phagocytic macrophages was measured. Data are presented as mean values ± SD out of *n* = 3 series of 10 confocal images for each condition. Statistical analysis was performed using a two‐tailed paired *t*‐test. *P < 0.05, **P < 0.01, ***P < 0.001. (B) Left: Confocal microscopy images of phagocytosis of BT474 cells. The white arrows show the phagocytic macrophages (red) having engulfed BT474 cell(s) and harbouring a green or yellowish colour. Scale bar represents 50 µm. Right: Details of a phagocytic macrophage (red) at higher magnification (60× oil immersion). Since BT474 are poorly adherent, nonphagocytosed free‐BT474 cells are washed away during the washing step. Scale bar represents 10 µm.

We further evaluated multimer‐directed CDCP using effector monocyte‐derived M2‐macrophages on BT474 tumour cells (Fig. 8A,B). Used individually, both FHR4/V_H_H(T) multimers and trastuzumab induced about 10% phagocytosis above control serum indicating that the phagocytosis taking place through Fc‐FcγRs interactions (ADCP) was as efficient as the phagocytosis taking place through C3b breakdown products, which is in accordance with previous data (Lee *et al.*, 2017). In contrast, pertuzumab and the FHR4/V_H_H(P) multimers did not elicit macrophage‐mediated phagocytosis. The combination of the two FHR4 multimers [FHR4/V_H_H(T) + FHR4/V_H_H(P)] or the mAbs (trastuzumab + pertuzumab) did not further increase phagocytosis. The highest percentages of phagocytic macrophages were observed when combinations of (a) pertuzumab with [FHR4/V_H_H(T)] (40% phagocytosis); or (b) trastuzumab with [FHR4/V_H_H(P)] (33% phagocytosis) were applied. In Fig. 8B left, the picture depicts phagocytic macrophages that are marked by arrows. One or more green‐stained BT474 cells are clearly visible within the red‐stained phagocytic macrophages. Figure 8B right depicts a magnification of a phagocytic macrophage with its blue nucleus, which shows one or more degraded BT474 cells including debris visible in green.

### 3.5 Solution‐phase complement activation

To determine whether the FHR4‐heteromultimeric immunoconjugates could spontaneously activate complement in the extracellular fluid phase before they reach their targets, we analysed the immunoconjugate‐mediated soluble phase complement using a CH_50_ assay (Fig. S6). When sheep erythrocytes previously sensitised with a rabbit haemolytic anti‐sheep erythrocyte serum are incubated with serum, the complement CP is activated and haemolysis takes place. If the complement in the serum is absent, the CH_50_ will be zero; if one or more complement components of the CP (including C3) are decreased, the CH_50_ will be proportionally decreased. Figure S6A depicts the titration of the serum and the calibration curve for the CH_50_ assay, with a dilution 1/50 in PBS to reach 75% lysis of sensitised sheep erythrocytes as an optimised condition for the CH_50_ assay. Figure S6B shows that the CH_50_ for the control aggregated IgG is 0.24 g·L^−1^, while the CH_50_ for the FHR4‐multimers was 0.55 g·L^−1^ (36.66 mg·kg^−1^). Most therapeutic antibodies are given at doses < 10 mg·kg^−1^ (Ryman and Meibohm, 2017), which corresponds to 0.15 g·L^−1^. At this concentration, the soluble phase complement activation using FHR4‐heteromultimeric immunoconjugates was slightly above that of the therapeutic antibodies (76% vs 86% haemolysis, respectively), indicating that the FHR4‐heteromultimeric immunoconjugates should not significantly active the solution‐phase complement at therapeutic concentrations.

## 4 Discussion

In the present study, we showed for the first time that immunoconjugates expressing multimeric complement‐activating FHR4 and anti‐HER2 anchoring moieties were able to lyse targeted HER2‐expressing breast tumour cells *in vitro* through CDC using the activation of the AP. We hypothesised that enhanced local densities of FHR4 at the surface of target cells are the critical point that would allow to overcome a CIT at the tumour cell surface that is established by the mCRP densities but also by bound FH. We therefore used the C4bp C‐terminal of the α and β chains of the multimerising scaffold (Dervillez *et al.*, 2006; Oudin *et al.*, 2000) to generate trifunctional heteromultimers, covalently linking (a) multimeric FHR4; to (b) trastuzumab‐ or pertuzumab‐competing nanobodies targeting HER2; and to (c) a monomeric eGFP allowing for the selection of molecular species with high FHR4‐valences.

We showed here that high FHR4 valence is the key element to trigger efficient AP activation (Figs 2D, 4C,D and 7A) and to induce MAC binding (Figs 5C and 7B,C), CDC (Figs 5A and S4B) and phagocytosis (Fig. 8A) on cells expressing HER2. Local densities of FHR4 on target plasma membranes thus determine the efficacy of counteracting the regulatory effect of FH and subsequent AP activation efficacy (Figs 2A,D, 4C,D and S4AB). Only molecular species enriched with sufficient FHR4 densities through sequential clonal selection and His‐Trap selective multistep purification succeed in overcoming FH‐established CIT on HER2‐tumour cells (Fig. 2D). A linear increase of FHR4 densities leads to the AP amplification loop taking place as ‘switch off/on’ biphasic reaction until the CIT is overcome (Fig. 2D). Multivalent FHR4 expression enhanced FHR4‐mediated FH deregulation and directed local AP activation which led to massive C3b deposition on target cell surface. The oligomerisation of FHRs is a common feature for FHR1, FHR2 and FHR5, which display a dimerisation motif in their N termini allowing the formation of homo and/or heterodimers as well as well as tetrameric complexes (van Beek *et al.*, 2017; Goicoechea de Jorge *et al.*, 2013; Jozsi *et al.*, 2015). This oligomerisation dramatically enhances FHR functional avidity *in vivo* as competitive antagonists of FH. FHR4, however, lacks this dimerisation motif. The natural homo‐dimerisation of FHR4 to a very low extent might, however, also exist, as previously reported (Hellwage *et al.*, 1999), although it has not been shown *in vivo*. FHRs compete with FH for C3b, iC3b and C3d binding, but, in contrast with FH, FHRs do not have a strong complement inhibitory effect due to the lack of FH‐like regulatory domains (Jozsi *et al.*, 2015). In particular, FHR4A lacks FH‐like decay accelerating activity for assembled C3bBb C3 convertases, but may affect C3b sensitivity to inactivation by factor I (Hebecker and Jozsi, 2012). FHR4 is unlikely to be of major physiological relevance (Hellwage *et al.*, 1999) because reported FHR4 plasma concentrations – 25.4 µg·mL^−1^ measured by Hebecker and Jozsi (2012) and 2.55 µg·mL^−1^ measured by Pouw *et al. *(2018) using FHR4A‐specific mAbs – are much lower than those of FH. Under physiological conditions, FHR4 may slightly reduce FH binding on late apoptotic, necrotic or senescent cells, thereby allowing enhanced C3b deposition and subsequent clearance of these opsonised cells by host phagocytes. Furthermore, FHR4 may preferentially bind to dead cell or pathogen surface *via* its interaction with C‐reactive protein (Hebecker *et al.*, 2010; Mihlan *et al.*, 2009), thus weakening FH binding and/or increasing complement activation on such surfaces.

Alternative pathway activation and C3b depositions are tightly regulated by soluble‐ and membrane‐associated complement regulators that (a) prevent the C3/C5 convertase assembly (C4bp, FH, CD46 and CD55); (b) accelerate the dissociation of membrane‐bound C3/C5 convertases (CD55); and (c) prevent the assembly of the terminal complement complexes C5b‐9 (CD59) (Jozsi *et al.*, 2015). Overexpression of some mCRPs is therefore considered to be a poor prognostic factor in cancer, whereas increased iC3b on tumours is a favourable prognostic factor (Liu *et al.*, 2014). Using three different HER2‐tumour cell lines being more or less resistant to complement attack, we showed that mCRP and HER2 expression patterns (Fig. 6) further determine a second level of control on AP expansion and lytic MAC densities, controlling FHR4‐multimer‐mediated CDC efficacy (Fig. 5). The most favourable situation was found for the SK‐OV3 cell line that displays low mCRP and high HER2 densities (Figs 5, 6 and S4). We further showed that the combination of the two optimised FHR4‐high valence multimers recognising two distinct HER2‐epitopes further enhanced mean FHR4 densities on cell surfaces and subsequent AP activation and cell death (Figs 5 and S4). When using the most complement‐resistant tumour cell line SK‐BR3, only the combination of the two optimised FHR4‐high valence multimers succeeded in eliciting CDC and, to a lesser extent, the combination of pertuzumab and the FHR4 multimers with trastuzumab‐competing V_H_H [FHR4/V_H_H(T)] (Fig. 5A). Complement‐mediated cell killing of breast, lung and ovarian tumour cells by combined trastuzumab and pertuzumab was previously shown to be efficient only when all three CD46, CD55 and CD59 mCRPs were suppressed using the siRNA gene silencing technology (Mamidi *et al.*, 2013). A comparative analysis of AP activation by the two combined FHR4‐multimers vs the two combined anti‐HER2 therapeutic mAbs clearly showed that at same levels of C3b deposition, subsequent MAC densities are far greater and CDC is up to four times higher when using combined FHR4‐multimers than when using combined mAbs (Figs 5 and S4). These data reinforce previous data hypothesising that the convertases associated with FHR4 are at least in part protected from FH‐mediated decay when compared to non‐FHR4‐associated convertases (Hebecker and Jozsi, 2012). By contrast, the formed convertases during mAb‐mediated CP activation are not protected from FH‐ and mCRP‐mediated decay, which leads to sublytic MAC binding and absence or weak CDC, despite initial massive C3b deposition.

We also confirmed that FHR4‐heteromultimeric immunoconjugates do exclusively activate AP using the analysis of C3b and/or C4d deposition on SK‐OV3 cells in serum conditions where AP, CP or both AP + CP were functional (Fig. S5). These results validated our hypothesis that FHR4‐heteromultimers, by competing with FH, subsequently activate the C3b amplification loop (AP) and thus have no role in the CP activation. In contrast, combined trastuzumab and pertuzumab activate the CP, which may have a retro‐control effect on the AP activation (Fig. S5), since more C3b deposition and less C4d deposition were observed when AP activity was recovered by the addition of FB. The advantage of using AP‐complement activators such as FHR4 is that they are not under the control of additional complement regulators of upstream pathways such as C1‐INH of C4bp that regulate CP.

To note, the control V_H_H(T) heteromultimer (lacking FHR4) displayed a certain degree of cytotoxicity that was independent from CDC, displaying a cell killing with no C3b deposition in the presence of NHS (Figs 5A,B and S4A,B), or displaying the same cell killing in the presence of decomplemented serum (Fig. 5A), and this effect was more pronounced on BT474 cells (Fig. 5A). The higher degree of V_H_H valences of these multimers in the absence of FHR4 moieties could explain their enhanced ability to cross‐link HER2 receptors on target cell surfaces, when compared to FHR4/V_H_H(T) or FHR4/V_H_H(P) multimers. It has previously been reported that HER2 cross‐linking prevents cells from forming signalling‐competent dimers with any EGFR family member, subsequently preventing any kinase dimerisation, and thus leading to a complete loss of HER2‐signalling and having an anti‐proliferative effect (Jost *et al.*, 2013). Various human tumour cell lines rely on HER2 signalling for their survival, referred to as ‘HER2‐addicted’ cells (Moasser, 2007). This may explain the additional cytotoxic effect of the V_H_H(T) multimer. A confocal microscopy analysis showed the homogeneous membrane distribution of FHR4/V_H_H(T) eGFP‐positive multimers on BT474 cells and a remarkable colocalisation with C3b deposition (Fig. 7A). In contrast, C5b‐9 staining is more disseminated, depicting intense red spots through and on the plasma membranes that are more distant from FHR4‐multimers (Fig. 7B,C).

Importantly, for potential therapeutic application, nonspecific complement activation by FHR4‐heteromultimeric immunoconjugates in the extracellular fluid is not desired as long as the multimers are not bound to their targets (Fig. S6). Based on a CH_50_ *in vitro* immunoassay, our results indicate that FHR4‐heteromultimeric immunoconjugates do not mediate significant soluble phase complement activation at concentrations that are commonly used in the clinic for therapeutic mAbs (Fig. S6B, blue area).

We then investigated the ability of opsonised BT474 tumour cells – upon multimer‐mediated complement activation – to be phagocytosed by monocyte‐derived M2‐macrophages, a phenomenon known as complement‐dependent cell‐mediated phagocytosis (CDCP) (Chan *et al.*, 2009; Gelderman *et al.*, 2004) (Fig. 8). We showed that FHR4/V_H_H(T) multimers elicit the phagocytosis of BT474 cells with the same efficacy than trastuzumab (Fig. 8A). Moreover, we showed that FHR4/V_H_H(T) multimers were superior to FHR4/V_H_H(P) to support the phagocytosis of BT474 cells, which correlates with the fact that FHR4/V_H_H(T) multimers elicit stronger C3b deposition than their FHR4/V_H_H(P) multimeric counterparts. Thus, a higher C3b deposition allowed increased phagocytosis. The combination of the mAbs or FHR4‐multimers with mAbs led to enhanced phagocytosis efficacy, particularly the combination of FHR4/V_H_H(T) heteromultimers and pertuzumab (Fig. 8A). This increased phagocytosis efficacy could be explained by the concomitant interactions of Fc‐FcγRs and iC3b‐CD11b/CD18 between target cell surfaces and macrophages, which reinforce the strength of physical contact between BT474 and macrophages, favouring the macrophage‐mediated phagocytosis of BT474 cells. Beyond CDC and direct cell killing, our data are in line with the observation that phagocytosis of opsonised tumour cells represents a critical mechanism to eliminate undesired opsonised cells by CDCP (Gelderman *et al.*, 2004). The complement breakdown products on HER2 cells are recognised by the CD11b/CD18 (CR3) receptor complex on macrophages (Chan *et al.*, 2009; Gelderman *et al.*, 2004).

Taken together, the present study showed that the degree of FHR4‐multivalency within the immunoconjugates determines the ability to overcome the FH‐ and mCRP‐mediated CIT. The association of two FHR4 multimers binding to two distinct HER2 epitopes further enhances FHR4 densities and deregulation efficacy, in particular on HER2 tumour cell lines that are resistant to complement attack. Under physiological conditions, FH, by preventing C3‐convertase formation, maintains the density of C3b molecules on host surfaces (Harrison and Lachmann, 1980) below a critical threshold from where C3b amplification proceeds without control (Jozsi *et al.*, 2015). When FHR4 multimers do efficiently antagonise FH and neutralise the first level of control of the AP, overexpressed mCRPs exert an additional level of control that will limit at different degrees the lytic MAC densities. Combined FHR4 multimers, however, succeeded in leading to 60% in cell death in the case of the most resistant tumour cell line used in this study (Fig. 5A). These results are in line with previous work showing that the neutralisation of FH with a specific antibody resulted in increased C3 fragment deposition and up to threefold CDC of colorectal cancer cells (Wilczek *et al.*, 2008).

**Figure 9:**
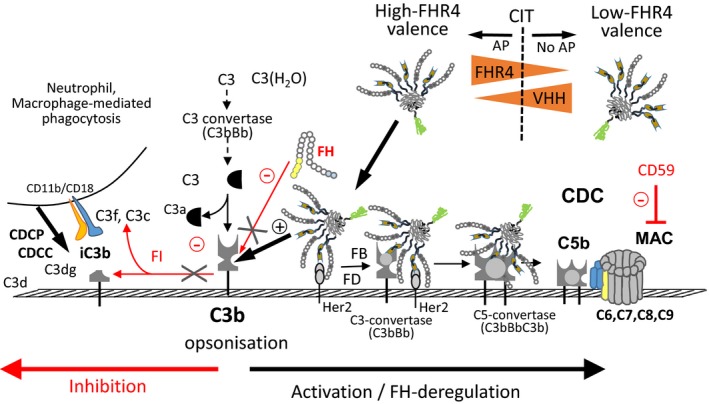
Mechanism of action of high FHR4‐valence immunoconjugates to activate AP on HER2 tumours cells and CDC. Optimised high FHR4‐valence heteromultimeric immunoconjugates, but not low‐FHR4 valence counterparts, activate C3b on target tumour cells by neutralising FH, allowing overcoming FH‐mediated CIT and by directly activating the AP by allowing the formation of the FHR4‐bound C3bBb convertases. Multivalent expression of FHR4 is key to allow local selective efficient competition with FH on tumour cell surfaces. Depending on mCRP overexpression pattern on target tumours, multimer‐mediated convertase formation and subsequent MAC densities can partially be controlled, but not prevented, leading in our hands from about 100% to 60% direct lysis according to the resistance of the cell to complement attack. Finally, C3b breakdown products on opsonised tumour targets are recognised by macrophages, allowing tumour cell clearance by CDCP. FI, factor I; FD, factor D.

Besides CDC, we showed that opsonised target tumour cells were phagocytosed by M2‐macrophages in a complement‐dependent manner. Multimeric FHR4 immunoconjugate‐mediated AP activation should therefore further potentiate the anti‐tumour response *via* complement receptors on different cell types (macrophages, NK cells), resulting in enhanced CDCC or CDCP and the recruitment and/or activation of effector cells. Figure 9 summarises the mechanism of action of FHR4 immunoconjugates. A limitation of this approach, like for all cancer immunotherapies, is the possibility of loss of HER2 overexpression overtime, either for primary tumours or for metastatic cells. It has been reported, however, that changes in HER2 expression actually seem to be extremely rare although a few cases have been reported, and the HER2 expression is stable between metastases and corresponding primary tumours (Carlsson *et al.*, 2004).

## 5 Conclusions

In conclusion, we describe in the present work an innovative strategy of destructive cell targeting consisting of locally neutralising FH in order to overcome FH‐mediated CIT and overactivating AP on target tumour cells. The few therapeutic mAbs whose activity lies in part in CDC are used in clinical practice to treat CLL and non‐Hodgkin’s lymphoma (Winiarska *et al.*, 2011). FHR4 multimeric immunoconjugates targeting CD20 – by replacing the anti‐HER2 targeting system – may therefore have a higher cytolytic potential in lymphoma cells than in solid tumour cells, whereas it has been shown that abrogation of the FH function in combination with ofatumumab or rituximab resulted in an increased susceptibility of primary CLL cells to CDC (Horl *et al.*, 2013).

## Conflict of interest

The authors declare no conflict of interest.

## Author contributions

CD designed the experiments and the manuscript, analyse the results and wrote the manuscript. JMP performed the AP activation assays, MAC binding and CDC assays, the confocal microscopy analyses and phagocytosis assays. CM designed and performed the surface plasmon resonance experiments. CV prepared the plasmids and produced the constructs. GI performed the flow cytometry analysis as well as western blots. MF produced and purified the multimers and performed the phagocytosis assays. MJ and JHMC contributed to the design of the experiments and reviewed the manuscript. XD designed the destructive cell targeting approach on tumour cells, the constructs of the immunoconjugates (patent PCT/EP2017/062283), designed the experiments and wrote the manuscript.

## Supporting information


**Fig. S1**. Expression of bifunctional and trifunctional heteromultimers and modulation of two multivalent functions relative with each other.
**Fig. S2**. Sequential cell sorting of original FHR4‐high valence multimer‐expressing cell clone (FHR4H) improved the mean FHR4‐valence within multimers.
**Fig. S3**. Improved multi‐step‐purification of FHR4/V_H_H(T) or FHR4/V_H_H(P) heteromultimeric immunoconjugates using His‐Trap columns and FPLC.
**Fig. S4**. (A, B) Flow cytometry analysis of FHR4‐multimer‐, control multimer‐ vs mAb‐mediated C3b deposits (A) and CDC (B) on BT474 tumour cells.
**Fig. S5**. Flow cytometry analysis of the complement pathways activated by FHR4‐heteromultimeric immunoconjugates.
**Fig. S6**. Analysis of fluid phase complement activation in NHS using a CH_50_ assay.
