# Supplementary material for: FHR4‐based immunoconjugates direct complement‐dependent cytotoxicity and phagocytosis towards HER2‐positive cancer cells
Source: Mol Oncol. 2019 Sep 30;13(12):2531–53. doi: 10.1002/1878-0261.12554 (PMC6887587; doi:10.1002/1878-0261.12554)
Supplement: Supplementary file 1 — Fig. S1. Expression of bifunctional and trifunctional heteromultimers and modulation of two multivalent functions relative with each other. Fig. S2. Sequential cell sorting of original FHR4‐high valence multimer‐expressing cell clone (FHR4H) improved the mean FHR4‐valence within multimers. Fig. S3. Improved multi‐step‐purification of FHR4/VHH(T) or FHR4/VHH(P) heteromultimeric immunoconjugates using His‐Trap columns and FPLC. Fig. S4. (A, B) Flow cytometry analysis of FHR4‐multimer‐, control multimer‐ vs mAb‐mediated C3b deposits (A) and CDC (B) on BT474 tumour cells. Fig. S5. Flow cytometry analysis of the complement pathways activated by FHR4‐heteromultimeric immunoconjugates. Fig. S6. Analysis of fluid phase complement activation in NHS using a CH50 assay. [file MOL2-13-2531-s001.docx]

**
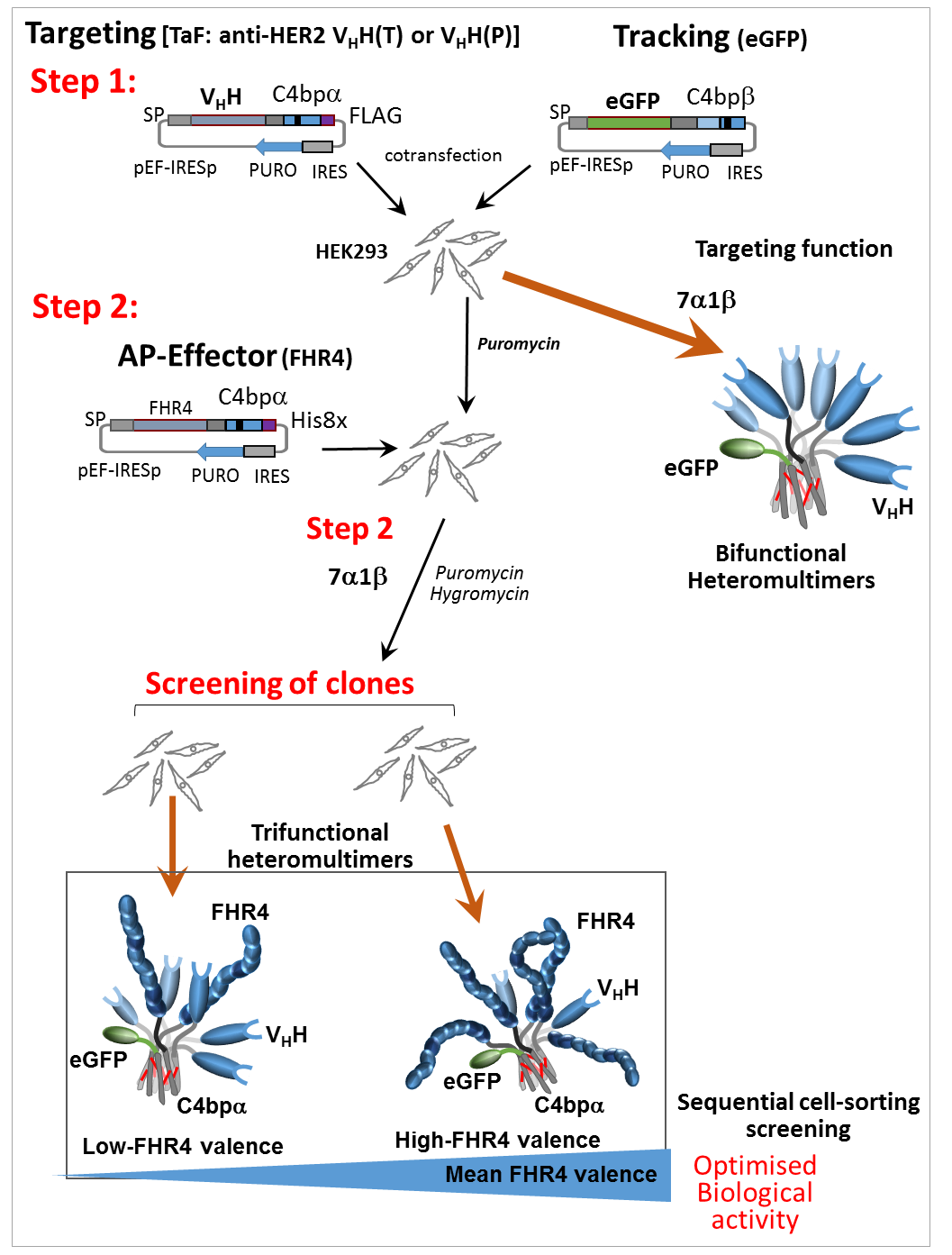
**

**Figure S1: Expression of bifunctional and trifunctional heteromultimers and modulation of two multivalent functions relative with each other: Step 1:** **Expression of the bifunctional heteromultimers** displaying (i) the multivalent targeting function and (ii) the monovalent eGFP tracking function. HEK293 cells were co-transfected with (i) the targeting and (ii) tracking function expression vectors. The targeting functions are fused to the C-terminal C4bp α-chain, followed by a FLAG tag, whereas the tracking function (eGFP) is fused to the C-terminal C4bp β-chain. Following stable co-transfection using puromycin selection, puromycin-resistant cell clones were isolated and cultured separately. Bifunctional multimer-containing supernatants from single clones were then analysed on target cells, by staining multimer-loaded cells with an anti-FLAG antibody. After a single-cell sorting and screening, the cell clone expressing the highest amount of bifunctional heteromultimers was further used in the second step. **Step 2: Expression of trifunctional heteromultimers** by adding an AP-effector function (FHR4) to the best bifunctional multimer-expressing clone. Selected clone from Step 1 was co-transfected with (i) the expression vector for FHR4 and (ii) pTK-Hygro for hygromycin selection. Further cell sortings and screenings were performed to select cell clones expressing high or low FHR4-effector valence trifunctional heteromultimers.


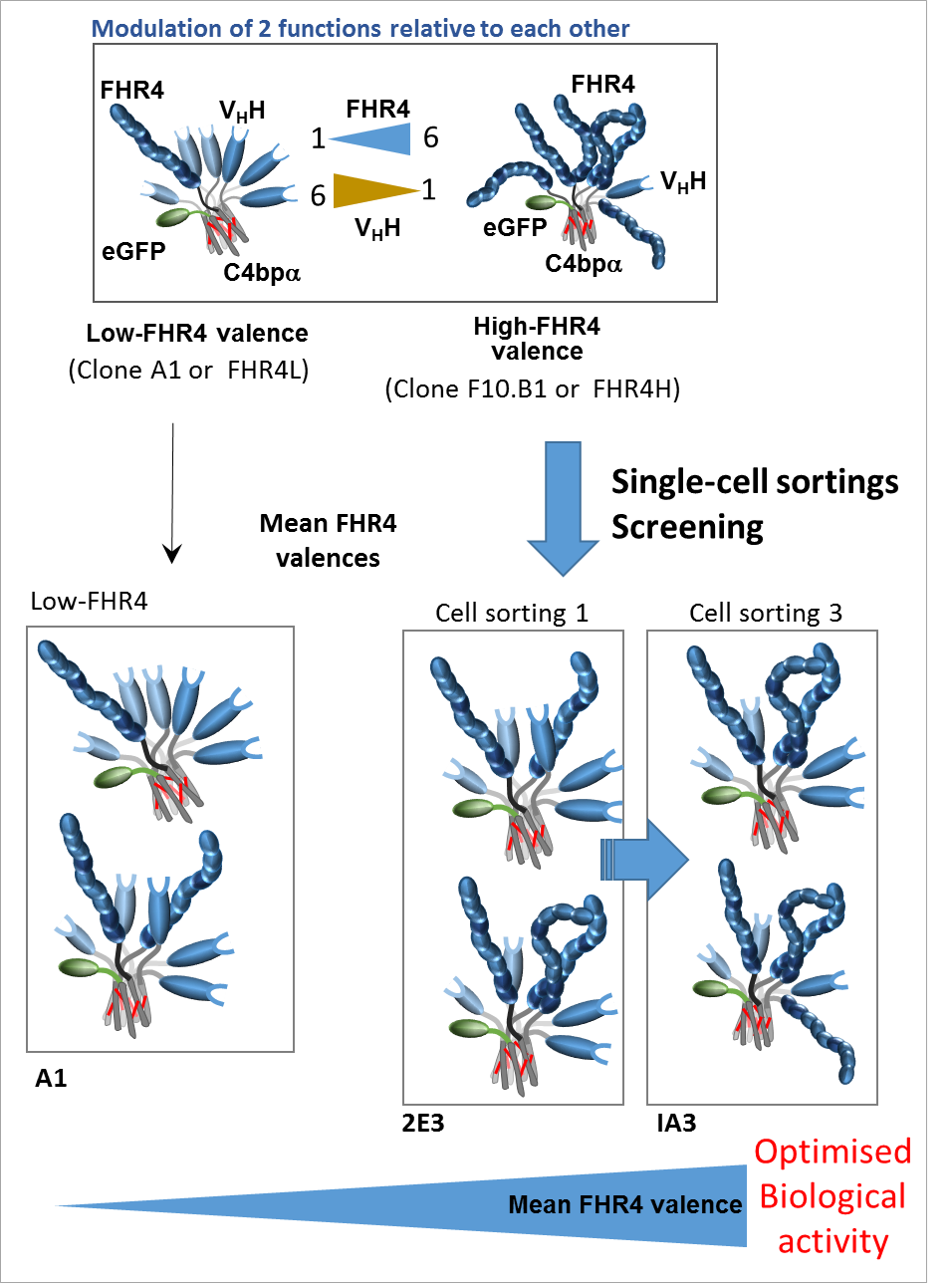


**Figure S2: Sequential cell sorting of original FHR4-high valence multimer-expressing cell clone (FHR4H) improved the mean FHR4-valence within multimers. a)** FHR4H-expressing cell clone was three times sequentially single-cell sorted. After each single-cell sorting, the cell clone expressing the highest mean FHR4-valence trifunctional heteromultimers was further single-cell sorted. Selected cell clones from the 3 single-cell sortings are 2E3, 2E9 and IA3, respectively. Successive single-cell sortings led to the selection of optimised cell clones with enhanced mean FHR4-valence multimer expression and optimised AP activity.

**
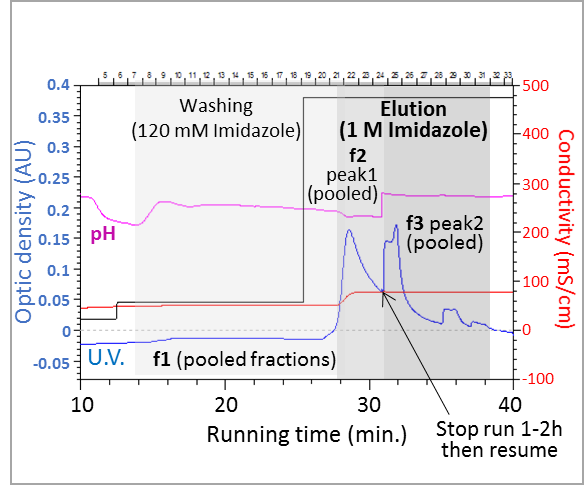
**

**Figure S3: Improved multi-step-purification of FHR4/V_H_H(T) or FHR4/V_H_H(P) heteromultimeric immunoconjugates using His-Trap columns and FPLC.** Detailed chromatogram representing the washing/elution steps, the latest consisting of an elution step using 120 mM imidazole, followed by a 2-step elution using 1M imidazole, with a 2-hour stop/resume step once the 2 peaks starts to decay. Higher FHR4-valence multimers display also more His tags, their release from the matrix by exchange with imidazole is favoured when the flow is stopped for a couple of hours.

**
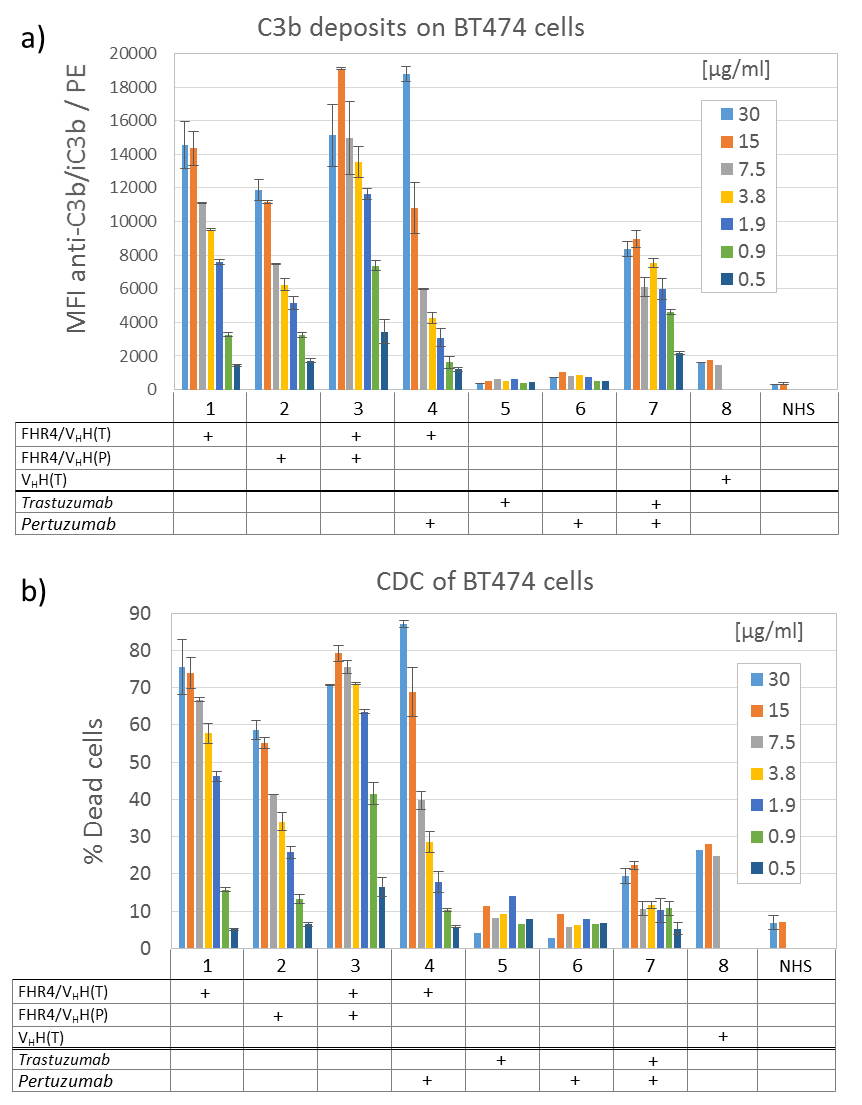
**

**Figure S4:** **a-b)** Flow cytometry analysis of FHR4-multimer-, control multimer- versus mAb-mediated C3b deposition (**a**) and CDC (**b**) on BT474 tumour cells. Two-fold serial dilutions starting at 30 µg/ml concentration of multimer or mAbs were used, and then incubated with 30% NHS for 30 min at 37°C. The multimers or antibodies were used either individually (1, 2, 5, 6, 8) or in combinations (3, 4, 7). V_H_H(T) control multimers (8) and NHS alone were used as negative controls. Cells were then stained with an anti-C3b mAb and the live/dead marker. Data are means +/- SDs of 3 experiments.

**
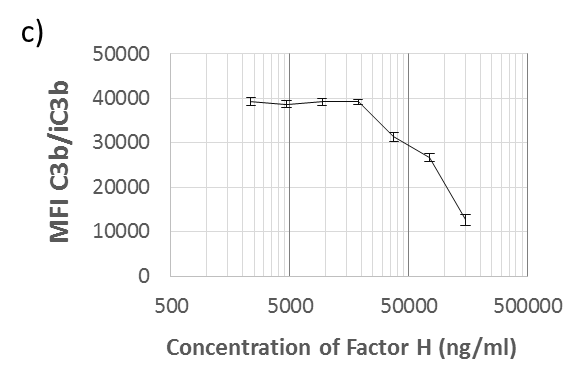
**

**Figure S4c:** Inhibition of FHR4/V_H_H(T) heteromultimer-induced C3b depositions on SK-OV3 cells by addition of serial concentrations of FH. SK-OV3 cells were incubated for 30 min at 37°C with constant concentration of FHR4/V_H_H(T) multimers (15 µg/ml) together with 2-fold serial dilutions of commercial human FH (starting from 150 µg/ml) in GVB^++^ supplemented with 25% C5-deficient human serum. Cells were then stained with an anti-human C3b/iC3b mouse mAb and analysed using flow cytometry.


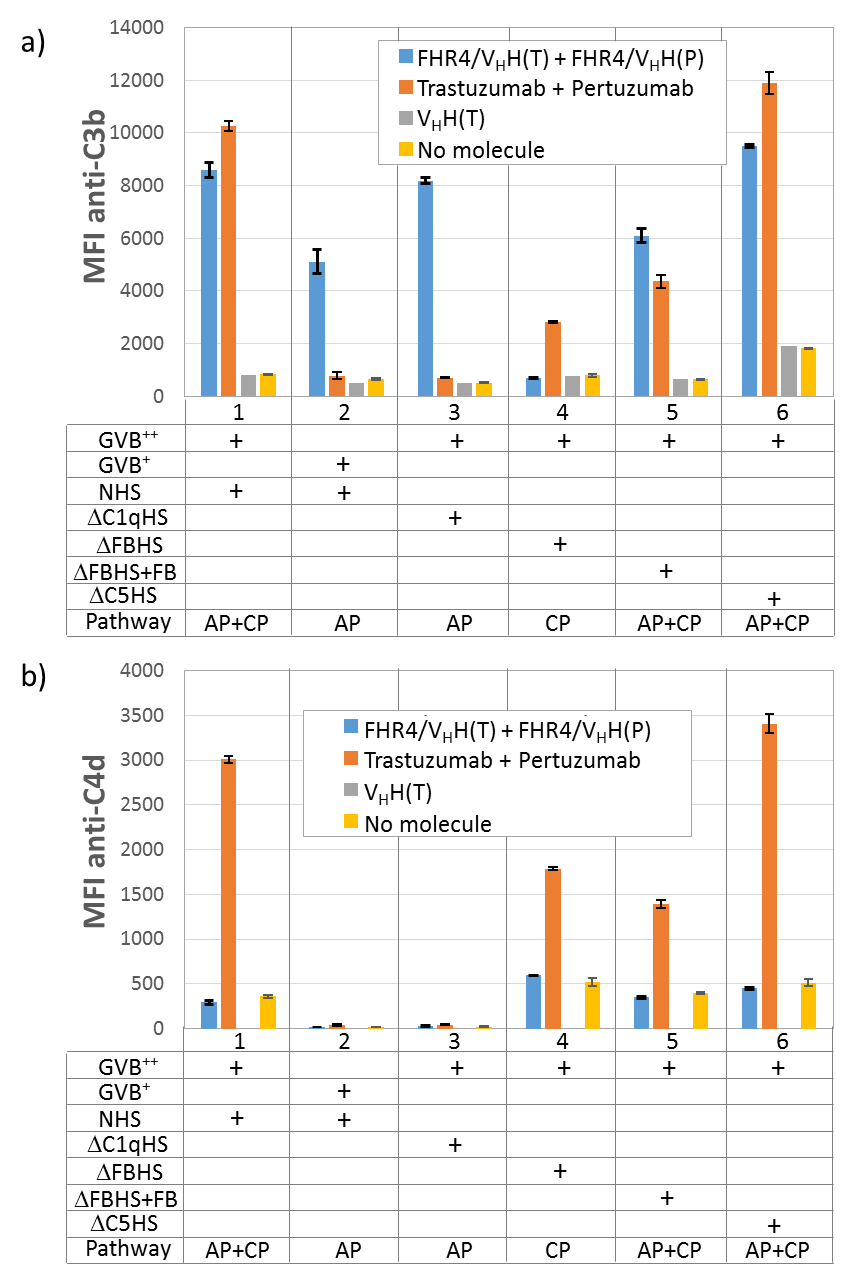
**Figure S5:** **Flow cytometry analysis of the complement pathways activated by FHR4-heteromultimeric immunoconjugates.** SK-OV3 cells were incubated with saturating concentrations (30 µg/ml) of (i) combined FHR4/V_H_H(T) and FHR4/V_H_H(P) multimers, (ii) combined trastuzumab and pertuzumab, (iii) V_H_H(T) control multimers or (iv) with no multimer/mAb (no molecule). Cells were then incubated with (1) 30%NHS/GVB^++^, (2) 30%NHS/GVB^+^, (3) 30% C1q-deficient human serum (ΔC1qHS), (4) 30% FB-deficient human serum (ΔFBHS), (5) ΔFBHS with 200 µg/ml FB or (6) 30% C5-deficient human serum (ΔC5HS) for 30 min at 37°C. **a)** SK-OV3 cells were analysed for C3b deposition. **b)** SK-OV3 cells were analysed for C4d deposition. Data are means +/- SD of 3 experiments.

**
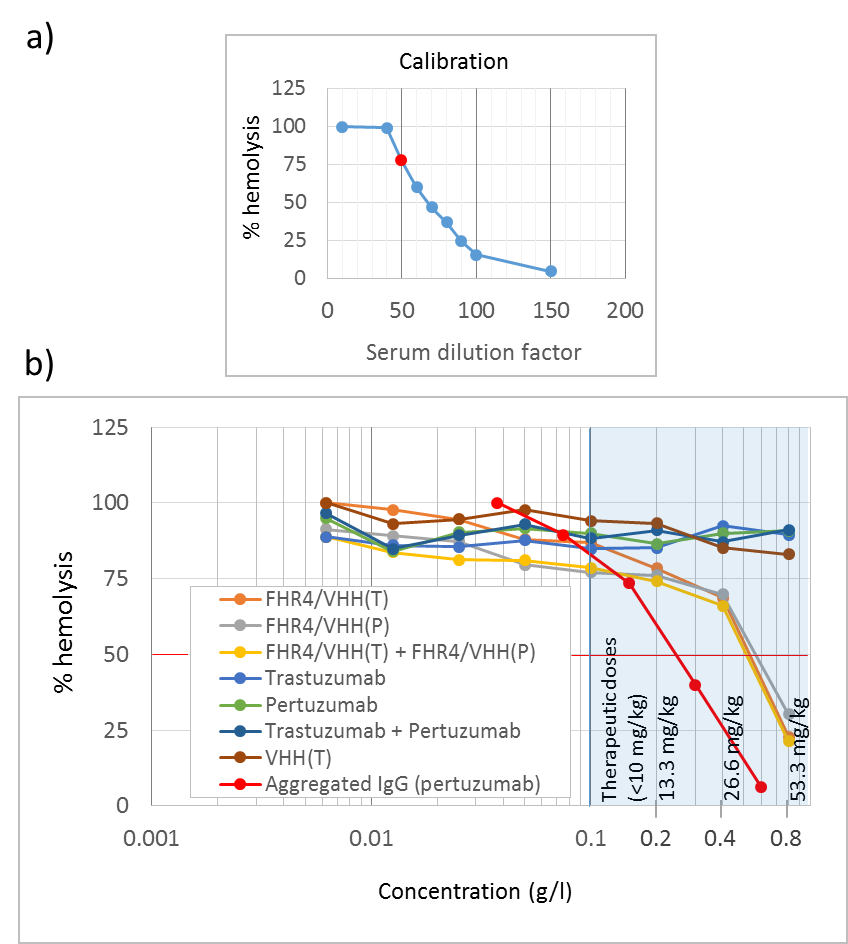
**

**Figure S6:** **Analysis of fluid phase complement activation in NHS using a CH_50_ assay. a) Serum titration:** Sensitized sheep erythrocytes (SSE) were incubated with serial dilutions (in PBS) of 90% NHS/10% GVB^++^ for 30 min at 37°C. A serum dilution of 1/50 (upper part of the linear curve) corresponding to 75% lysis of sensitised sheep erythrocytes was used as endpoint for optimal sensitivity of hemolytic complement activity (red dot). **b)** **CH_50_ assay:** Two-fold serial dilutions of FHR4-heteromultimers, mAbs and V_H_H(T) control multimers (starting concentrations were 0.8 g/l) or positive control (aggregated IgG) were incubated in 90% NHS/GVB^++^ for 1h at 37°C. Then, serums diluted 1/50 in PBS were incubated with SSE for 30 min at 37°C. The OD of collected supernatants was measured at 416 nm and the percentage of hemolysis for each dilution was then calculated. Most of the therapeutic antibodies are used at concentrations < 10 mg/kg (0.15 g/l). The blue area corresponds to the “off range” antibody concentrations that are superior to those used in the clinic.
